# Characterization of bacterial diversity associated with a Salar de Atacama native plant *Nitrophila atacamensis*

**DOI:** 10.1186/s40793-025-00766-7

**Published:** 2025-08-06

**Authors:** Leonardo Zamora-Leiva, Jorge Soto, Celián Román-Figueroa, Francisca Peña, Luciano Univaso, Manuel Paneque

**Affiliations:** 1 Bionostra Chile Research Foundation, Almirante Lynch 1179, San Miguel, Santiago, 8920033 Chile; 2 Department of Environmental Sciences and Natural Resources, Faculty of Agricultural Sciences, Universidad de Chile, Santa Rosa 11315, La Pintana, Santiago, 8820808 Chile

**Keywords:** Rhizosphere, Salar de atacama, Hydrogeological systems, Aguas de quelana, Soncor, Pir4 lineage, *Aliifodinibius*, *Candidatus* tremblaya

## Abstract

**Background:**

The rhizosphere microbiota is vital for the modulation of plant growth and adaptation, especially in extreme environments. *Nitrophila atacamensis* is an endemic and endangered plant species in the Salar de Atacama, Chile. However, the specific relationships between *N. atacamensis* and its microbiota remain largely unknown. We analyzed the bacterial communities in the rhizosphere and bulk soils associated with *N. atacamensis* across eight sites, including Aguas de Quelana and Soncor, which are two distinct hydrogeological systems.

**Results:**

We used high-throughput sequencing of the 16S rRNA gene to classify 886 different bacterial genera from 13,138 unique operational taxonomic units, distributed between rhizosphere and bulk soil samples. Microbial composition and diversity differed significantly between the rhizosphere and bulk soils. The microbial clustering observed among the Aguas de Quelana sites was based on their bacterial profiles and not their physicochemical properties, whereas the Soncor system exhibited high microbial heterogeneity. These findings suggest a potential role for water dynamics in shaping bacterial communities in Aguas de Quelana. Furthermore, the rhizosphere samples clustered into three distinct clades based on microbial composition; Pir4 lineage, *Aliifodinibius*, and *Candidatus* Tremblaya genera dominated specific clades.

**Conclusions:**

This study provides the first comprehensive characterization of the rhizosphere and bulk soil bacterial diversity associated with *N. atacamensis*, providing important ecological and functional insights into its microbial interactions. This further highlights the importance of understanding the rhizosphere microbial diversity in extreme environments and its potential implications for biotechnological applications and conservation efforts. Our findings provide a foundation for future research on microbial–plant interactions in arid ecosystems.

**Supplementary Information:**

The online version contains supplementary material available at 10.1186/s40793-025-00766-7.

## Background

The Atacama Desert is located in northern Chile. It is the driest desert worldwide and one of the most extreme ecosystems on Earth [1]. It extends over an approximately 1,000 km-long stretch bound by the Pacific Ocean on the west and the Andes Mountains on the east. This stretch presents extreme environmental conditions, such as low barometric pressure, high solar and ultraviolet radiation, minimal precipitation, high salinity, and strong winds [1]. These extreme characteristics have forced the species inhabiting the region to develop unique adaptations, resulting in biodiversity of high scientific value [2].

The most relevant ecosystems in this desert are the salt flats, an extremely fragile ecosystem. These are hydrogeological formations characterized by hypersaline lagoons, and wetlands with abundant microbial and plant communities [2]. The Salar de Atacama is among the largest evaporite basins on the planet. Its surface area is approximately 3,000 km² [3]. This salt flat includes a complex hydrogeological system comprising brackish water, salt flats, brine, underground recharge flows from the Andes, and evaporative discharge processes in marginal areas [3]. It has several wetlands on Salar’s eastern edge, divided into the Soncor, Aguas de Quelana, and Peine-Tilopozo hydrological systems. The Soncor system comprises three permanent lagoons: Puilar, Chaxa, and Barros Negros, and large areas of seasonal flooding adjacent to the Puilar and Barros Negros lagoons. Aguas de Quelana is a sector punctuated by seasonally flooded surfaces of small extensions; the Peine and Tilopozo sector comprises three permanent lagoons: Salada, Saladita, and Interna [4]. Due to water scarcity, these hydrological systems are key water provider regions, allowing biodiversity and ecosystem services in arid environments and hosting unique organisms, including *Nitrophila atacamensis*, a xerophytic plant endemic and restricted to the Salar de Atacama. *N. atacamensis* grows in fragile environmental conditions, such as saturated soil close (but without direct contact) to the water table, highly prone to structural loss and compaction. This limits the soil gas exchange, water storage, and conduction capacities, subsequently reducing root development [5–7]. This plant grows in loamy textured soils, with high salinity levels (concretions or saline accumulations on the surface) and moist soil profiles without contact with the water Table [7]. Its location has been described only on the eastern edge of the Salar de Atacama, in the Soncor and Aguas de Quelana hydrological systems [8]. Moreover, *N. atacamensis* is highly affected by climatic change, accentuated by anthropogenic activities such as the construction of roads and brine extraction [8], which makes it highly relevant from a conservation point of view.

Climate change will make soil increasingly arid, conditions to which desert plants have already adapted [9]. They have developed specific adaptations such as Crassulacean Acid Metabolism photosynthesis and modified leaf structures. Desert plants also enable microorganisms to thrive in dry, nutrient-poor soils. Thus, bioprospecting of desert plants such as *N. atacamensis* has enormous biotechnological potential because of specific adaptations to extreme conditions, but mainly due to the microorganisms that inhabit the rhizosphere and endosphere. These microorganisms can increase nutrient input and plant resistance to drought and heat stress [10]. Plant-associated bacterial communities in the rhizosphere are crucial for plant adaptation and productivity as they facilitate nitrogen fixation, nutrient recycling, and resistance to environmental stresses [11]. Bacterial genera commonly coexist with plants, favoring their growth (such as *Bacillus*). Additionally, some common microorganisms among plants have been reported in different extreme environments, such as the Atacama Desert and Antarctica [12]. However, just as plants develop specific adaptations to particular environmental conditions, microorganisms are also uniquely suited to specific climates. This creates a niche for bioprospecting bacteria adapted to salinity and drought, which can be identified for biotechnological purposes [13]. Plant–bacteria interactions are crucial for the survival of plant species in the hypersaline environments of the Salar de Atacama [14]. However, the specific relationships between *N. atacamensis* and its microbiota remain largely understudied, and the alterations induced by environmental physicochemical and geographical conditions in the microbiota remain unclear.

In this study, we aimed to characterize bacterial communities associated with the rhizosphere of *N. atacamensis* and compare them with those of the surrounding soil at eight sites at the Salar de Atacama. Additionally, we sought to elucidate the factors shaping these communities by analyzing microbial diversity and its relationship with the physicochemical variables in two different hydrogeological systems: Aguas de Quelana and Soncor. This study contributes to a broader understanding of the microbial biodiversity in *N. atacamensis* rhizosphere and its potential role in the plant’s resilience to its extreme thriving conditions.

## Methods

### Study site and sample collection

**Fig. 1:**
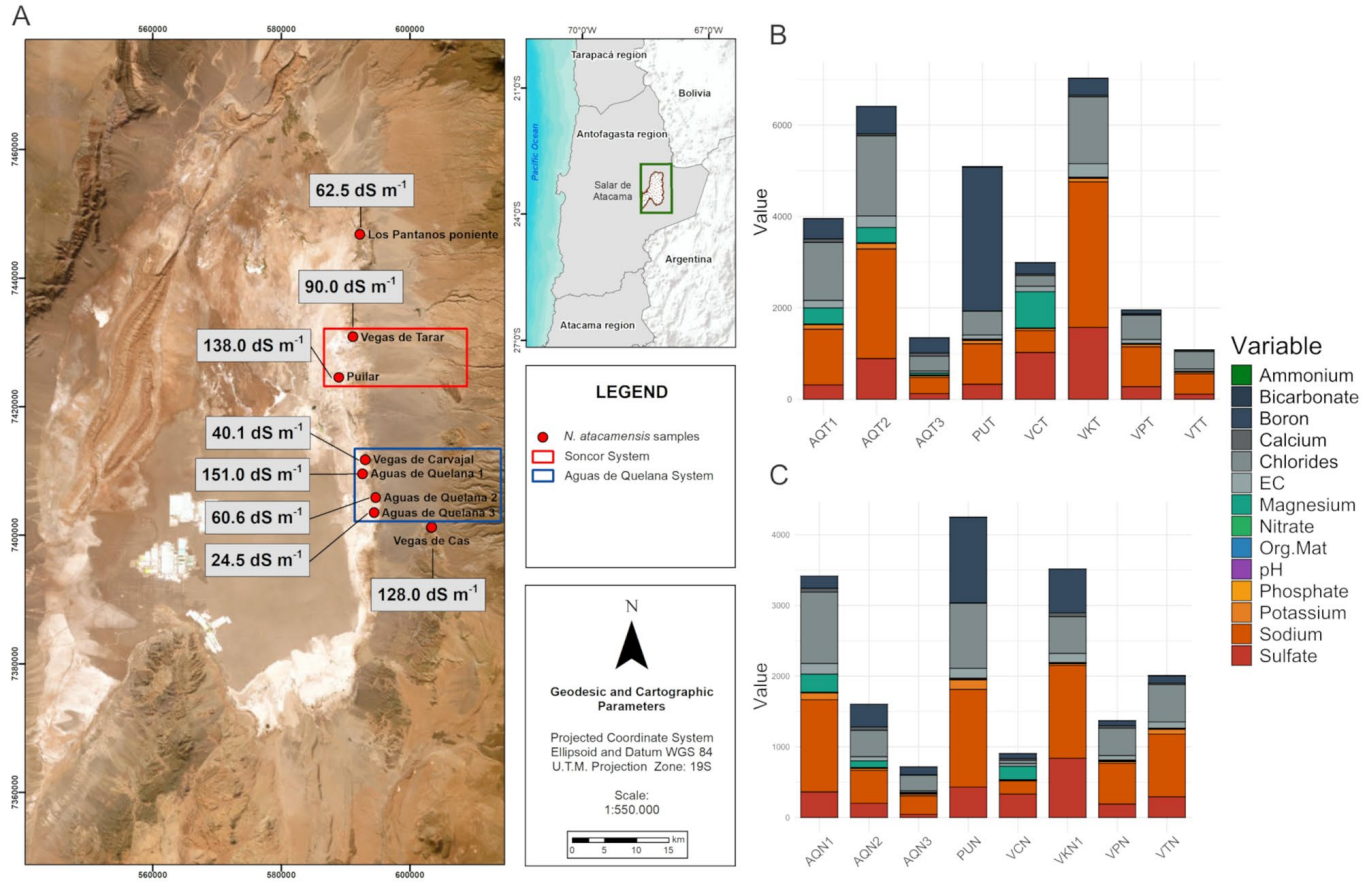
Nitrophila atacamensis location and physicochemical characterization of the soil and rhizosphere samples. (**A**) The study area comprises a system of lakes and wetlands. The eight lakes and wetlands are outlined in four inserts, where the hydrogeological systems are shown in red (Soncor system) and blue (Aguas de Quelana system). Physicochemical characterization of (**B**) bulk soil and (**C**) rhizosphere sampling sites. Sample abbreviations: Aguas de Quelana 1 (AQN1 and AQT1); Aguas de Quelana 2 (AQN2 and AQT2); Aguas de Quelana 3 (AQN3 and AQT3); Puilar (PUN and PUT); Vegas de Cas (VKN and VKT); Vegas Carvajal (VCN and VCT); Vega Los Pantanos Poniente (VPN and VPT); and Vegas Tarar (VTN and VTT)

The study area is located at the Salar de Atacama, Antofagasta Region, Chile (Fig. 1). Three field surveys were conducted: first (July 20–23, 2022), second (December 13–15, 2022, and third (March 09–11, 2023). The samples were collected from eight sites in the interior of the Salar de Atacama: Vega Los Pantanos Poniente, Vega Tarar, Vega Puilar, Vega Carvajal, three sites of Vega Aguas de Quelana, and Vega de Cas. All sites are located on the eastern slope of the Salar.

Individual *N. atacamensis* plants were extracted with their root systems. These were shaken to remove excess soil not adhering to the roots. The plants were stored in 50 mL Falcon™ tubes (Thermo Fisher Scientific, Waltham, MA, USA) and transported to the laboratory. Plants were vortexed to dislodge soil particles adhering to the roots, and the soil was subsequently used for DNA extraction. Simultaneously, the soil removed from around the roots (within 3 cm) was collected as a composite rhizosphere soil sample for physicochemical analysis.

The composite soil samples comprised five subsamples of approximately 200 g, which were combined and mixed to better represent the sample area. Subsequently, the composite soil samples were used for chemical analysis of the following parameters: pH, electrical conductivity, as well as PO_4_³⁻, SO_4_²⁻, NO_3_⁻, Cl⁻, NH_4_⁺, Mg, K, Na, B, Fe, Mn, Cu, Zn, and NaHCO_3_ contents [15, 16]. Total organic matter (OM) was estimated using the formula: OM (%) = 1.72 × total organic carbon (%).

For bulk soil, composite samples were obtained 20 m from the sampled *N. atacamensis* plant, in a location free of vegetation. These samples were used for physicochemical analysis and DNA extraction.

To cluster the sampling sites based on physicochemical variables, a Euclidean distance matrix was generated using the dist function from the “stats” v4.3.2 package in R. This distance matrix was then used to perform hierarchical clustering with the hclust function, applying the “Ward.D2” agglomeration method from the “stats” package.

### DNA extraction and sequencing

The rhizospheric and bulk soil samples contain complex matrices for DNA extraction because of their high salt concentrations and low microbial abundance. Thus, a procedure combining two extraction methodologies [17, 18] was developed to extract DNA efficiently from the sample obtained from the Salar de Atacama. To determine microorganism concentration and salt removal, 2.5 g of soil was suspended in 50 mL of cell extraction buffer [17] and shaken overnight. The suspension was centrifuged at low speed (1,000 rpm for 5 min), and the supernatant was filtered through a 0.2-µm membrane [18]. The membranes were cut into small pieces and subjected to a lysis buffer supplemented with 25 µL of proteinase K (20 µg/mL). Microbial DNA was extracted using the DNeasy PowerSoil Kit (Qiagen, Mo Bio Laboratories, Hilden, Germany). The tubes were shaken and incubated at 65 °C for 1 h, followed by DNA purification using the kit reagents and a column system. Given the low microbial biomass of the samples, negative extraction controls were included to verify that the recovered microbial DNA originated from the soil itself and not from potential contamination introduced by reagents or materials used during the extraction process.

The extracted DNA was quantified using a QUBIT™ 4 fluorometer (Thermo Fisher Scientific, Waltham, MA, USA). The integrity of the 16 S rRNA region was confirmed by amplification (approximately 1,500 bp) via conventional polymerase chain reaction (PCR) with the following primers: 27 F (5′-AGAGTTTGATCCTGGCTCAG-3′) and 1492R (5′-GGTTACCTTGTTACGACTT-3′) [19]. Each 25 µL PCR mixture contained 1.0 µL of template DNA, 1.0 µL of each primer (10 µM), 12.5 µL of 2× GoTaq^®^ Green Master Mix (Promega, Madison, WI, USA), and 9.5 µL of ddH_2_O. PCR conditions were as follows: primary denaturation at 95 °C for 5 min; followed by 30 cycles of: 95 °C for 60 s, 55 °C for 60 s, and annealing at 72 °C for 90 s, followed by a final extension at 72 °C for 10 min. PCR was performed using an ESCO Swift MaxPro thermal cycler (Thermo Fisher Scientific). DNA quality in the samples was verified using 1% agarose gel electrophoresis performed using the SYBR™ Safe DNA gel stain (Thermo Fisher Scientific) coupled to a 1-kb molecular weight marker. Electrophoresis was performed at 100 V for 30 min, and the bands were visualized in a transilluminator chamber. Rhizosphere and bulk soil samples were considered suitable for sequencing only when both yielded successful amplification of the full-length 16S rRNA gene, and when no amplification was observed in either the DNA extraction blanks or the PCR negative controls.

The preparation of genomic libraries and subsequent sequencing of the V3–V4 variable regions of the 16 S rRNA gene were performed by Macrogen Inc. (South Korea) on the MiSeq^®^ sequencer (Illumina). Adapters were added to construct the libraries, and bacteria-specific primers were used to amplify the V3–V4 hypervariable regions (341 F: 5′-CCTACGGGGGNGGCWGCAG-3′ and 805R: 5′-GACTACHVGGGTATCTAATCC-3′). The Illumina 16 S library preparation protocol was applied [20]. Each 25 µL PCR mixture contained 2.5 µL of template DNA, 5.0 µL of each primer (1 µM), and 12.5 µL of 2× KAPA HiFi HotStart ReadyMix (Roche Sequencing Solutions, Pleasanton, CA, USA). The PCR conditions included primary denaturation at 95 °C for 3 min; followed by 25 cycles of: 95 °C for 30 s, 55 °C for 30 s, and 72 °C for 30 s; and a final extension at 72 °C for 5 min. PCR products were validated using the Bioanalyzer 2100 system (Agilent Technologies, Santa Clara, CA, USA), and sequencing was performed using a 2 × 300-bp paired-end configuration.

### Bioinformatic analysis

The FASTQ files were processed using Qiime v2023.9.1 [21]. Read quality was evaluated using FastQC v0.11.9 [22]. Based on this evaluation, the adapters were removed, and the lengths of the forward and reverse sequences were trimmed to 270 and 205 bases, respectively, using the DADA2 de-noise-paired plugin [23]. Quality was maintained using PHRED 20 [21]. Taxonomic assignment was performed using the classify-sklearn plugin in the SILVA database version 138.1 (https://www.arb-silva.de) [24]. New unified sequences (operational taxonomic units [OTUs] with 99% identity) were aligned using MAFFT [25], and the maximum likelihood was inferred using FastTree [26].

The abundance, taxonomy, phylogeny, and metadata of the OTUs were integrated into a phyloseq object for subsequent analyses using the Phyloseq v1.46.0 package (PhyloSeq) [27] in R v4.3.2 (https://www.r-project.org). The quality control filters described by Callahan et al. [28] were used: samples with < 1,000 reads were excluded and unassigned OTUs were removed. The mean number of reads per taxon was > 1 × 10^− 5^, and OTUs not observed more than twice in at least 10% of the samples were excluded. Additionally, samples were rarefied to 10,000 reads, such that all samples had the same read count. Then, a phyloseq object was used to calculate alpha and beta diversities, and the Microeco v1.4.0 package was used to construct relative abundance plots [29]. OTUs were transformed into proportions, and relative abundance was used to measure the composition of prokaryotic communities at the phylum and genus levels. Relative abundances were used to generate an initial dendrogram with the clustering_plot function to evaluate potential similarities between the samples. Alpha diversity was calculated using different indices such as the Chao1 (estimate of richness by species) [30], Pielou (estimate of abundance and uniformity of OTUs in the samples) [31], Shannon (entropic information on abundance of observed OTUs) [32], and Simpson (dominance) [33] indices. To determine beta diversity, all samples were compared according to the sampling points using seven matrices: Robuts Aitchinson, Bray–Curtis, Canberra, Jaccard, Jensen-Shannon Divergence (JSD), Unifrac, and wUnifrac. Additionally, the differential abundance of taxa was analyzed by implementing linear discriminant analysis effect size (LEfSe, LDA value > 2, α = 0.05) [34] supported with an Analysis of Compositions of Microbiomes with Bias Correction 2 (ANCOM-BC2) analysis [35], using the Microeco v1.4.0 package.

Redundancy analysis (RDA) was performed using the Microeco v1.4.0 package to evaluate the effect of soil physicochemical parameters on bacterial community composition. Before the analysis, all environmental variables were included as predictors in a forward and backwards selection procedure using the ordistep function in the *vegan* v2.6-4 package, starting from a null model and iteratively adding variables that significantly improved the model fit based on permutation tests (*n* = 10,000). The final RDA model was constructed at the genus level (relative abundance > 1%) using only the environmental variables selected as significant (magnesium, bicarbonate, chlorides, nitrate, calcium, pH, and potassium). The significance of the overall model and individual canonical axes was assessed using an ANOVA-like permutation test with 10,000 permutations. Furthermore, the Spearman index was utilized with the Microeco v1.4.0 package for correlation analysis of taxa (genus level) associated with physicochemical variables.

## Results

### Chemical characterization

In the present study, we characterized the bacterial communities in soil samples associated with *N. atacamensis* populations at various sites across the Salar de Atacama, Antofagasta, Chile (Fig. 1 and Supplementary Table 1). Sampling was performed along the eastern side of the Salar de Atacama at every spot where the plant was spotted during the expedition. In total, eight sites were sampled: Vega los Pantanos Poniente (VPN for rhizosphere; and VPT for bulk soil); Vegas Tarar (VTN for rhizosphere; and VTT for bulk soil) and Puilar (PUN for rhizosphere and PUT for bulk soil), both sites from the Soncor hydrogeological system; three Aguas de Quelana lagoons, named Aguas de Quelana 1, 2 and 3 (AQN1, AQN2, and AQN3 for rhizosphere; and AQT1, AQT2, and AQT3 for bulk soil), Vegas de Carvajal (VCN for rhizosphere and VCT for bulk soil), four sites from Aguas de Quelana hydrogeological system; and Vegas de Cas (VKN for rhizosphere and VKT for bulk soil) (Fig. 1). The sampling sites were characterized to determine the physicochemical characteristics of both bulk and rhizospheric soils (Supplementary Table 1). In general, all samples were extremely saline with electrical conductivities (ECs) ranging from 24.5 dS/m to a maximum of 297 dS/m; slightly alkaline with pH values ranging from 7.61 to 9.12 (with the exception of Vegas Tarar bulk soil, VTT, which was slightly acidic at pH 6.89). In terms of composition, some samples showed a markedly high composition of ions such as sulfates, chlorides, sodium, and boron. AQT2 sample (Aguas de Quelana 2 bulk soil) contained 893 mg/L of sulfates, 1,753 mg/L of chlorides, 2,395 mg/L of sodium, and 604 mg/L of boron, whereas VKT (Vegas de Cas bulk soil) contained 1,572 mg/L of sulfates, 1,467 mg/L of chlorides, 3,184 mg/L of sodium, and 369 mg/L of boron. The VKT sample showed 9.68 mg/L of nitrites, which was six times higher than that in the AQT3 (Aguas de Quelana 3 bulk soil) sample, showing the second-highest concentration of this ion. This resulted in extremely high EC values (255 dS/m for AQT2 and 297 dS/m for VKT). In terms of organic matter, the values ranged from 0.25 to 5.82%, and the rhizosphere samples generally showed higher organic matter content than in the bulk soil samples. Aguas de Quelana 3 showed 3.99% organic matter in the rhizosphere (AQN3) and 5.82% in the bulk soil (AQT3), which was higher than the average of 1.48% estimated in the two types of samples. Moreover, when clustering the sampling sites according to their physicochemical characteristics, no pattern was discerned in terms of geographical location or specific hydrogeological system. The Aguas de Quelana 1 and 2 sites clustered together but belonged to the same clade as that of Vegas de Cas (the southernmost point), whereas the remaining sites of the Aguas de Quelana system (Aguas de Quelana 3 and Vegas Carvajal) clustered with the sites of the Soncor system and Vega los Pantanos Poniente samples (Supplementary Fig. 1).

### Microbial diversity and taxonomic assignment

Microbial DNA was obtained from the 24 rhizosphere and bulk soil samples. The V3–V4 regions of the 16 S rRNA gene were sequenced and subjected to filtration and quality control. We obtained 2,805,079 sequences (average, 58,386; median, 54,102). Taxonomic assignments were made at the phylum (98.18%), class (89.47%), order (89.54%), family (85.17%), and genus (80.63%) levels.

**Fig. 2:**
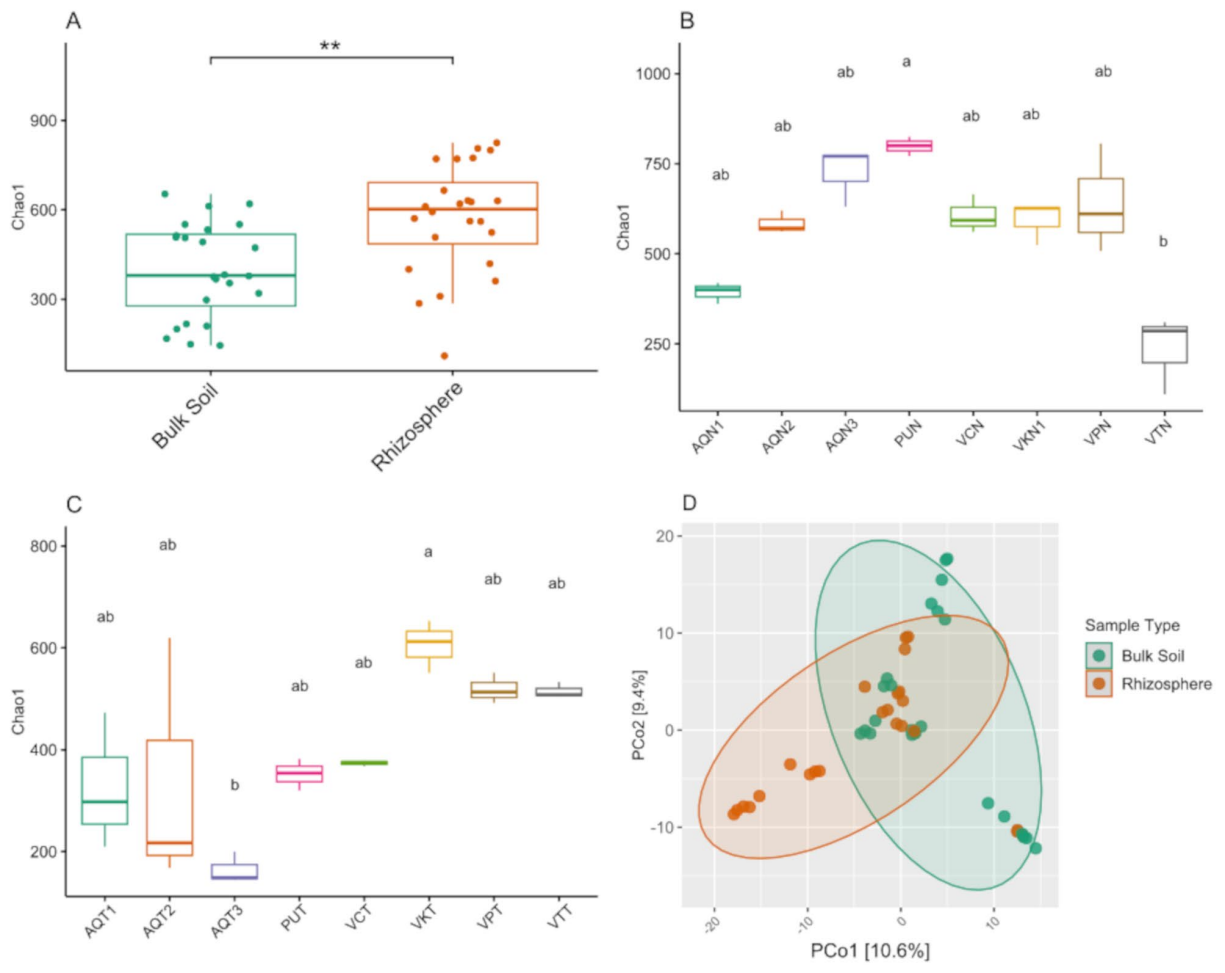
Diversity indexes for the rhizosphere and bulk soil samples. (**A**) Alpha diversity for *Nitrophila atacamensis* rhizosphere and bulk soil based on the Chao1 index (Wilcoxon test, ** < 0.01). (**B**) Alpha diversity for all rhizosphere soil samples and (**C**) bulk soil samples, based on the Chao1 index (Kruskal–Wallis test, *p* < 0.05). (**D**) Beta diversity for *Nitrophila atacamensis* rhizosphere vs. bulk soil samples, as determined using the Robust Aitchinson test (PERMANOVA, *p* = 9.999e-05). Sample abbreviations: Aguas de Quelana 1 (AQN1 and AQT1); Aguas de Quelana 2 (AQN2 and AQT2); Aguas de Quelana 3 (AQN3 and AQT3); Puilar (PUN and PUT); Vegas de Cas (VKN and VKT); Vegas Carvajal (VCN and VCT); Vega Los Pantanos Poniente (VPN and VPT); and Vegas Tarar (VTN and VTT)

First, we evaluated the compositional differences between the rhizosphere soil of *N. atacamensis* and bulk soil. We found significant differences in the alpha diversity indices between these two samples (Wilcoxon test, *p* < 0.05; Fig. 2A), indicating differences in bacterial richness. A comparison of the alpha diversity of all rhizosphere samples showed high similarities, except for the Puilar (PUN) and Vegas Tarar (VTN) samples, both of which belong to the Soncor hydrogeological system and exhibit differences (Kruskal–Wallis, *p* > 0.05, Fig. 2B). Comparison of the same index for the bulk soil samples showed similar results, but with differences between Aguas de Quelana 3 (AQT3), Vegas de Cas (VKT), and Vegas Tarar (VTT) (Kruskal–Wallis, *p* > 0.05, Fig. 2C). Beta diversity analysis showed significant differences between the rhizosphere and bulk soil, suggesting that the communities were not the same in both ecosystem types (Wilcoxon test, *p* < 0.001; Fig. 2D).

### Comparative analysis of rhizosphere and bulk soil bacterial diversity

**Fig. 3:**
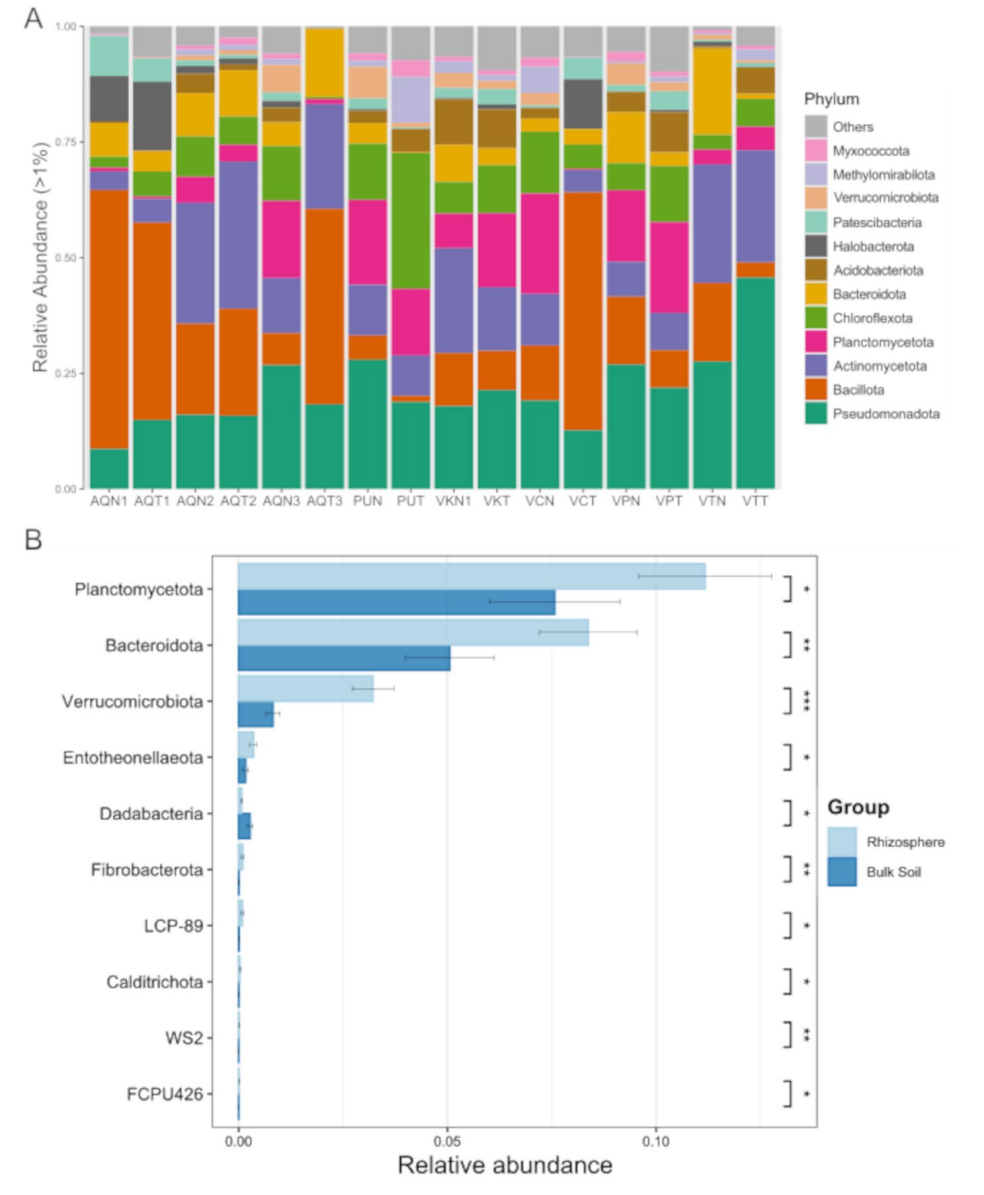
Relative abundance and differential abundance at the phylum level. (**A**) Relative abundances of 10 most abundant bacteria per sample at the phylum level, and (**B**) differential abundance of phyla with differences between rhizosphere and bulk soil samples (LEfSe, α = 0.05; Krustall–Wallis, * = *p* < 0.05, ** = *p* < 0.01, *** = *p* < 0.001). Sample abbreviations: Aguas de Quelana 1 (AQN1 and AQT1); Aguas de Quelana 2 (AQN2 and AQT2); Aguas de Quelana 3 (AQN3 and AQT3); Puilar (PUN and PUT); Vegas de Cas (VKN and VKT); Vegas Carvajal (VCN and VCT); Vega Los Pantanos Poniente (VPN and VPT); and Vegas Tarar (VTN and VTT)

The bacterial composition across all samples included 50 phyla. Of these, three were unique to the rhizosphere and three to bulk soil, whereas 44 were shared between both samples. They primarily included the phyla Pseudomonadota (21.34% and 21.18% of relative abundance, on average), Bacillota (17.87% and 22.58%), Actinomycetota (14.99% and 14.90%), Planctomycetota (11.17% and 7.50%), Chloroflexota (8.01% and 9.39%), Bacteroidota (8.37% and 5.06%), Acidobacteriota (3.40% and 3.66%), Halobacterota (1.8% and 3.57%), Patescibacteria (2.31% and 2.38%), Verrucombiota (3.22% and 0.82%), Methylomirabillota (1.64% and 1.99%), and Myxococcota (1.16% and 1.03%), which showed > 1% relative abundance (Fig. 3A). Even the samples with almost similar relative abundance (on average) of some phyla (e.g., Pseudomonadota) showed relative abundances considerably higher than those observed at other sites. For example, Vegas Tarar bulk soil (VTT) showed a relative abundance of 45.68% for Pseudomonadota, which is more than double the average detected in the bulk soil samples. The Vegas Carvajal bulk soil (VCT), Aguas de Quelana 1 (rhizosphere and bulk soil; AQN1 and AQT1), and AQT3 (Aguas de Quelana 3, bulk soil) samples showed > 40% relative abundance of Bacillota, whereas the samples that did not belong to the Aguas de Quelana hydrogeographical system showed ≤ 17% relative abundance of this phylum. To evaluate these differences, we performed LEfSe analysis and identified 10 differentially abundant phyla between rhizosphere and bulk soil groups. The richness of Bacteroidetes, Planctomycetota, Verrucomicrobiota, Endotheonellaeota, and Fibrobacterota was significantly higher in the rhizosphere group than in the bulk soil group, whereas Dadabacteria, LCP-89, Calditrichota, FCPU426, and WS2 showed the opposite result (Fig. 3B).

**Fig. 4:**
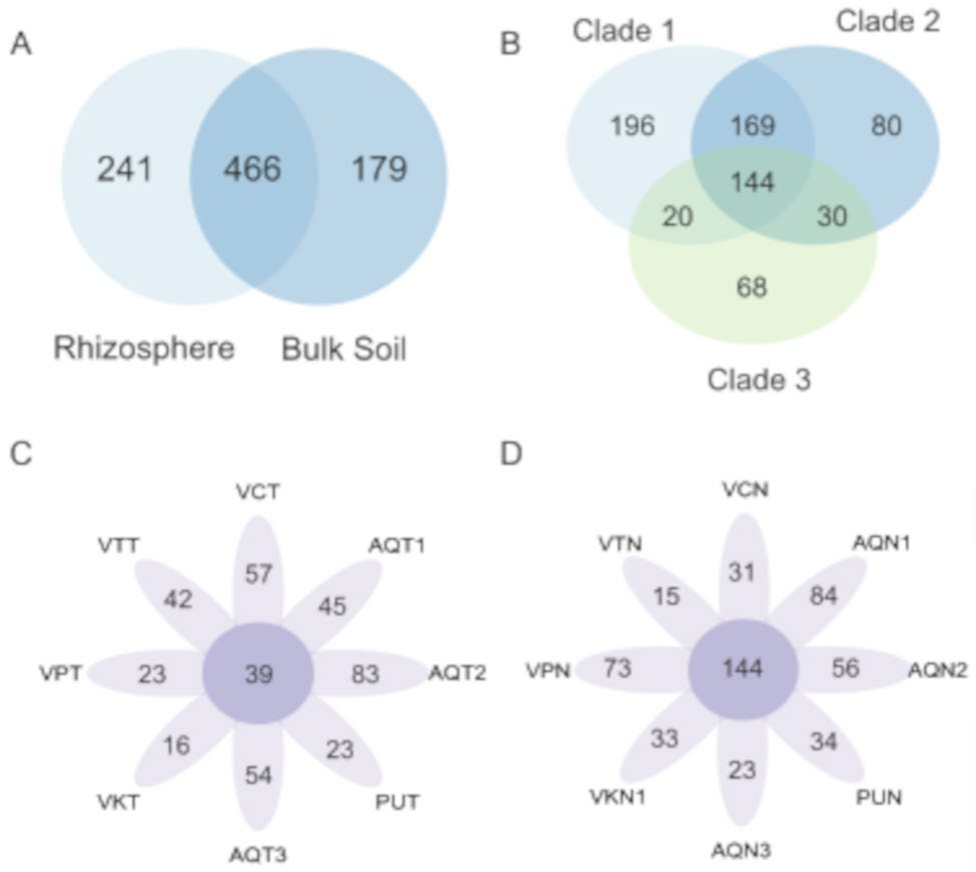
Venn and petal diagrams of genera shared for (**A**) rhizosphere and bulk soil samples, (**B**) the three rhizosphere clades. (**C**) all rhizosphere samples of *Nitrophila atacamensis*. (**D**) All bulk soil samples. Sample abbreviations: Aguas de Quelana 1 (AQN1 and AQT1); Aguas de Quelana 2 (AQN2 and AQT2); Aguas de Quelana 3 (AQN3 and AQT3); Puilar (PUN and PUT); Vegas de Cas (VKN and VKT); Vegas Carvajal (VCN and VCT); Vega Los Pantanos Poniente (VPN and VPT); and Vegas Tarar (VTN and VTT)

**Fig. 5:**
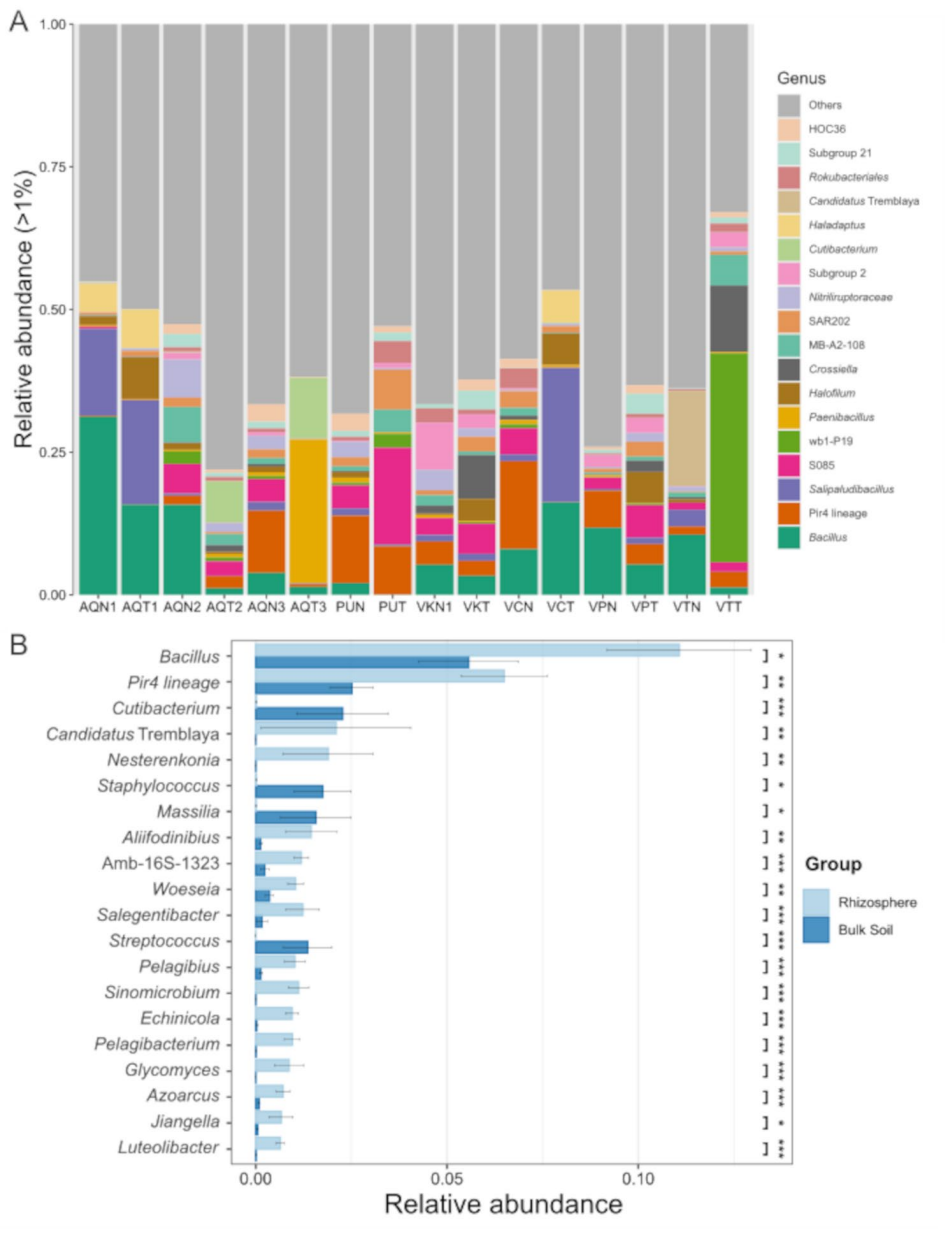
Relative and differential abundances at the genus level. (**A**) Relative abundances of the 20 most abundant bacteria per sample at the genus level, and (**B**) differential abundance of the first 20 genera with differences between rhizosphere and bulk soils (LEfSe, α = 0.05; Kruskal–Wallis, * = *p* < 0.05, ** = *p* < 0.01, *** = *p* < 0.001). Sample abbreviations: Aguas de Quelana 1 (AQN1 and AQT1); Aguas de Quelana 2 (AQN2 and AQT2); Aguas de Quelana 3 (AQN3 and AQT3); Puilar (PUN and PUT); Vegas de Cas (VKN and VKT); Vegas Carvajal (VCN and VCT); Vega Los Pantanos Poniente (VPN and VPT); and Vegas Tarar (VTN and VTT)

At the lower taxonomic level, 886 different genera were classified: 241 exclusively in the rhizosphere, 179 exclusively in bulk soil, and 466 shared between both sample types (Fig. 4A). Of these genera, 141 were shared for all rhizosphere samples (Fig. 4B) and 39 were shared for all bulk soil samples (Fig. 4C). The 20 most abundant bacterial communities (relative abundance > 1%) included *Bacillus* (11.07% and 5.56%), Pir4 lineage (6.49% and 2.52%), *Salipaludibacillus* (2.98% and 5.58%), S085 (3.06% and 4.02%), wb1-P19 (0.50% and 5.03%), *Paenibacillus* (0.46% and 3.42%), *Halofilum* (0.72% and 2.81%), *Crossiella* (0.42% and 2.83%), MB-A2-108 (1.56% and 1.60%), SAR202 clade (1.23% and 1.91%), *Nitriliruptoraceae* family (2.08% and 0.80%), *Acidobacteriae* Subgroup 2 (1.59% and 1.10%), *Cutibacterium* (0.01% and 2.27%), *Haladaptatus* (0.63% and 1.55%), *Candidatus* Tremblaya (2.10% and 0.00%), *Rokubacteriales* (1.10% and 0.95%), Acidobacteriota Subgroup 21 (0.78% and 1.27%) and HOC36 (1.25% and 0.78%) (Fig. 5A). The most abundant genus in the rhizosphere samples was *Bacillus*. Its abundance was higher in the rhizosphere soil than in bulk soil in seven of the eight samples. Vegas Carvajal (VCN) was the only site that showed the opposite trend (higher abundance of *Bacillus* in bulk soil than in rhizosphere). Furthermore, the 20 most abundant genera among these belonged to only eight phyla and 12 classes. Of these, three belonged to the phylum Bacillota and class Bacilli, and only *Bacillus* was more abundant in the rhizosphere, whereas *Salipaludibacillus* and *Paenibacillus* were more abundant in the bulk samples. A similar trend was observed in the phylum Pseudomonadota and class Gammaproteobacteria. Of the four detected genera, two were more abundant in bulk soil (wb1-P19 and *Halofilum*), whereas HOC36 and *Candidatus* Tremblaya showed the opposite trend. The latter was exclusive to the rhizosphere and almost exclusively from Vegas Tarar (VTN).

LEfSe analysis showed 162 differential genera between the two groups (Kruskal–Wallis, *p* < 0.05). The 20 genera with the highest relative abundance are shown in Fig. 5B. The richness of *Bacillus*, Pir4 lineage, *Candidatus* Tremlaya, *Nesterenkonia*, *Aliifodinibius*, *Sinomicrobium* (rhizosphere-exclusive), *Pelagibacterium*, *Echinicola*, Amb-16 S-1323, *Salengibacter*, *Pelagibius*, *Glycomyces*, *Woeseia*, *Luteolibacter*, *Azoarcus*, and *Jiangella* was significantly high in the rhizosphere group, whereas *Cutibacterium*, *Staphylococcus*, *Massilia*, and *Streptococcus* showed significantly high richness in the bulk soil group (Fig. 5B). Additionally, the genera *Planktosalinus*, *Chitinophaga*, *Kangiella*, *Methylophaga*, *Roseovarius*, *Isoptericola*, and *Chelativorans* were exclusively found in the rhizosphere with a relative abundance > 0.1%.

### Environmental factors influencing microbial communities

We performed a clustering based on relative abundance (at the phylum level) to evaluate the factors affecting the composition of microbial communities. This result showed that the bulk soil samples were grouped into two clades: one with all four samples from the Aguas de Quelana system, and another with the remaining four samples (Puilar and Vegas Tarar from the Soncor system; Vega los Pantanos and Vegas de Cas) (Supplementary Fig. 6).

Clustering of rhizosphere samples showed that microbial communities of *N. atacamensis* were not associated with geographical location or location inside the hydrogeological system (Fig. 6A). Based on this clustering, we defined three groups of samples: clade 1, Puilar, Aguas de Quelana 3, Vegas Carvajal, and Vega los Pantanos; clade 2, Vegas de Cas, Aguas de Quelana 2, and Vegas Tarar; and clade 3, Aguas de Quelana 1. This is supported by the beta diversity analysis results, which showed differences between the clades at the phylum (Fig. 6B) and genus levels (not shown), where differences were observed between clades 1 and 2 (pairwise post hoc test, *p* < 0.0001), clades 1 and 3 (pairwise post hoc test, *p* < 0.01), and clades 2 and 3 (pairwise post hoc test, *p* < 0.05).

**Fg. 6:**
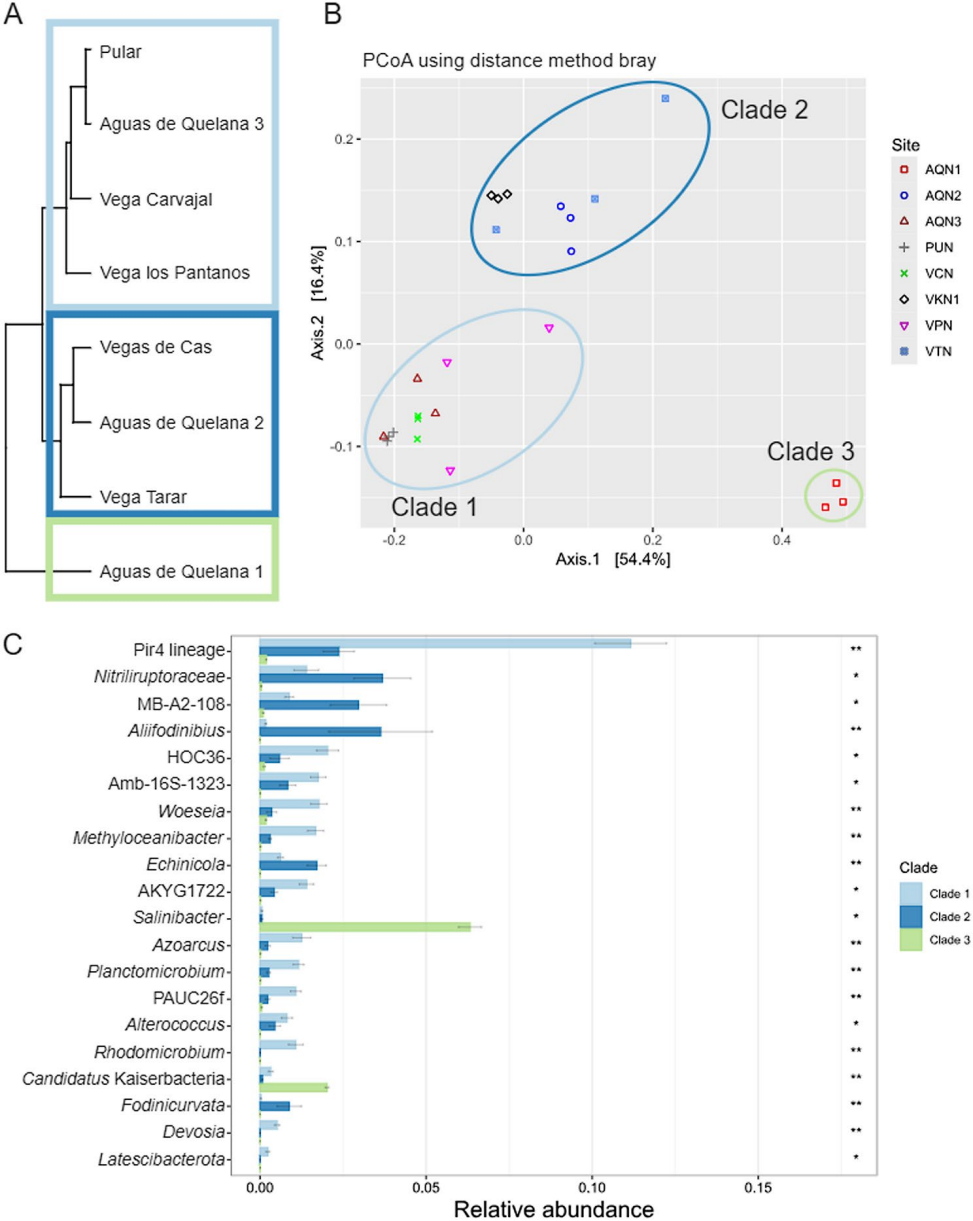
Clustering of rhizosphere samples, analysis of beta diversity, and differential abundance of rhizosphere clades. (**A**) Clustering of sampling sites based on relative abundance at the phylum level. (**B**) Principal coordinate analysis of Bray–Curtis distance at the genus level (PERMANOVA, *P* = 9.99991e-06). (**C**) Relative abundances of the 20 most abundant bacteria per sample at the genus level for the three rhizosphere clades. Sample abbreviations: Aguas de Quelana 1 (AQN1); Aguas de Quelana 2 (AQN2); Aguas de Quelana 3 (AQN3); Puilar (PUN); Vegas de Cas (VKN); Vegas Carvajal (VCN); Vega Los Pantanos Poniente (VPN); and Vegas Tarar (VTN and VTT)

To evaluate the variables that generated these differences, we analyzed the presence of differentially abundant taxa in the three clades. In total, 707 classified genera were identified among all three clades: 196 exclusive genera in clade 1; 80 exclusives to clade 2; 68 exclusives to clade 3; 20 genera shared between clades 1 and 3; 169 shared between clades 1 and 2; 30 shared between clades 2 and 3; and 144 shared between all three clades (Supplementary Fig. 6D). LEfSe analysis showed 134 differentially abundant genera among the clades, and the 20 with the highest relative abundance are shown in Fig. 6C. Some genera, such as *Methylophaga*, *Verrucomicrobium*, *Pseudorhodobacter*, *Vicinigus*, and *Dojkabacteria* (rhizosphere exclusive), were also overrepresented in one of the three clades.

**Fig. 7:**
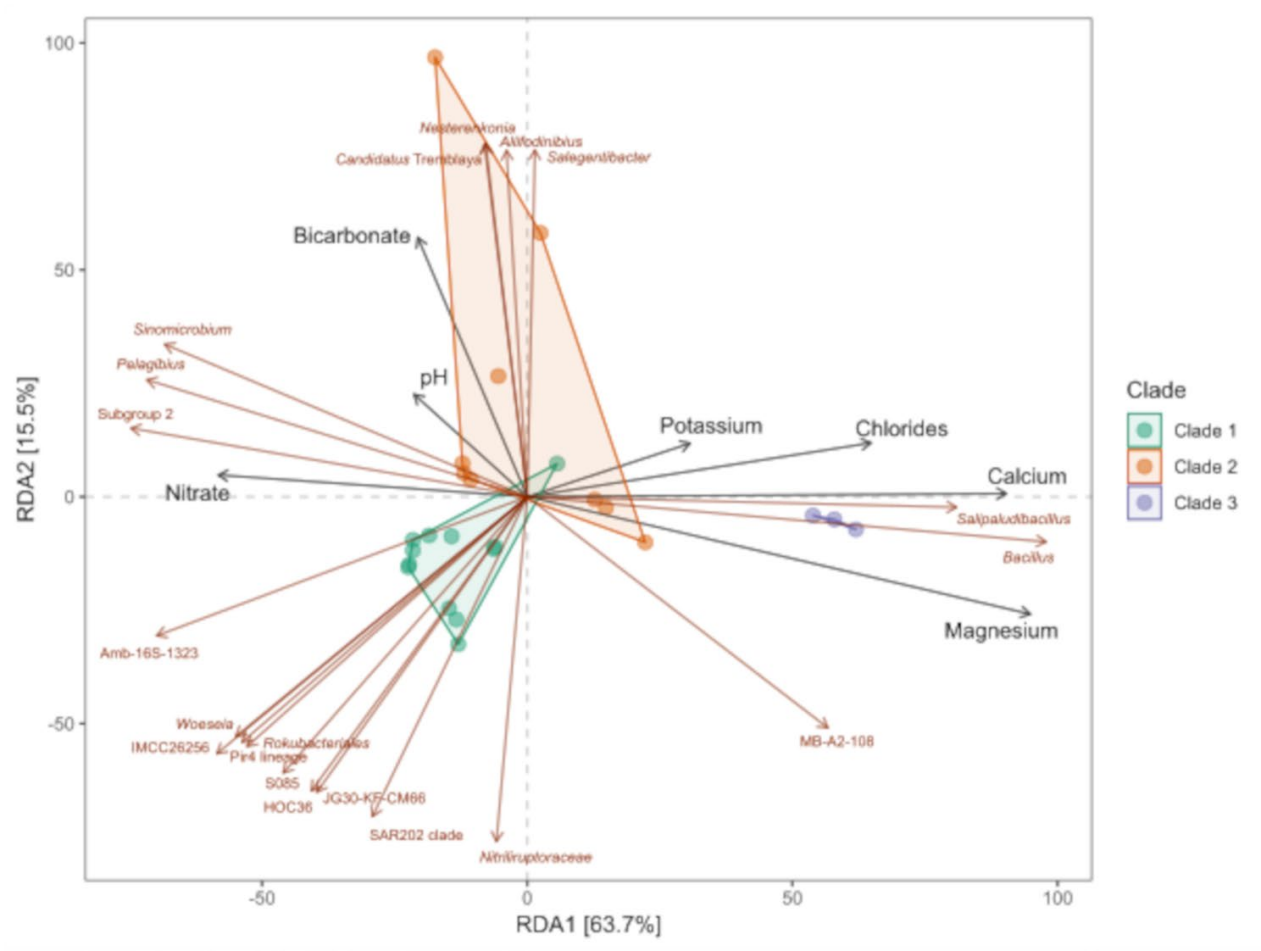
Redundancy analysis (RDA) of the distribution of most abundant genera with respect to seven environmental variables. Clades are indicated based on area: green for clade 1 (red dot and line), orange for clade 2 (blue dot and line), and purple for clade 3 (purple dot and line). Red arrows indicate the genera and their names; black arrows indicate environmental variables

We performed RDA including all physicochemical parameters (described in Fig. 1). All environmental variables were included as predictors in a forward selection procedure using the ordistep function, after which only seven physicochemical variables showed significance: magnesium, bicarbonate, chlorides, nitrate, calcium, pH, and potassium. The total inertia was 9.087e + 06, whereas the constrained inertia was 7.856e + 06, indicating that the constrained variables could explain 86.45% of the variation in the distribution of known genera. Finally, the first two components of the constrained eigenvalues exhibited proportions of 63.7% and 15.5%, respectively. The sampling points within the first component of the RDA were divided into two groups: the first group comprised clade 1 and half of the clade 2 samples, which were associated with nitrate, bicarbonate, and pH; the second group comprised clade 3 and the other half of the clade 2 samples, which were associated with potassium, chloride, calcium, and magnesium (Fig. 7).

The genera *Nesterekonia*, *Aliifodinibius*, *Candidatus* Tremlaya, *Sinomicrium*, *Pelagibius*, Subgroup 2, Amb-16 S-1323, *Woeseia*, IMCC2656, Pir4 lineage, *Rokubacteriales*, HOC36, JG30-KF-CM66, S085, SARR202, and *Nitriliruptoraceae* were positively associated with nitrate, bicarbonate, and pH, since their relative abundance increased with an increase in these parameter levels. *Salipaludibacillus*, *Bacillis*, and MB-A2-108 were positively associated with potassium, chlorides, calcium, and magnesium (Fig. 7). These results are consistent with the results of the Spearman correlation analysis for all measured physicochemical parameters and the relative abundance of the genus (Supplementary Fig. 11).

Overall, the groups formed by sample location based on physicochemical parameters (Fig. 7) were highly similar to those formed based on beta diversity analysis (Fig. 6B) and differential abundance (Fig. 6C). Clade 1, which contained low calcium concentrations, was closely related to the Pir4 lineage, HOC36, Amb-16 S-1323, and *Woeseia*. The genus *Aliifofodinibius* was primarily present in clade 2, and specifically, Vegas Tarar, which exhibited one of the highest pH values and lowest magnesium concentrations; these parameters were related positively and negatively, respectively, according to the RDA and Spearman correlation analysis.

## Discussion

### *N. atacamensis* rhizosphere presents higher diversity than bulk soil

This study showed the differences in the bacterial composition between the bulk soil and rhizosphere soil samples of *N. atacamensis* at Salar de Atacama. Unexpectedly higher diversity was observed in the rhizosphere than in the bulk soil samples. Generally, the rhizosphere selects microorganisms from the bulk soil [36, 37], translating to reduced microbial diversity in the roots. However, the rhizosphere of *N. atacamensis* shows the opposite trend, which is rare (but not unique) and is commonly observed in forest ecosystems [37] and rice plants [38]. For example, rice releases oxygen on its roots, which allows higher presence of aerobic bacteria in the rhizosphere compared with the rest of the nearby environment [38]. This indicates that environmental heterogeneities, such as root exudates, interact to produce selective effects in the rhizosphere, which are of special importance to specialized plants. *Ammodendron bifolium* shows a similar trend as that of *N. atacamensis*, where the rhizosphere community is more abundant and diverse than that of the bulk soil community at different soil depths [39]. The plant host provides nutrients for underground microbial communities through plant litter, root exudates [12, 40, 41], and strengthens microbial activity [42]. However, the bacterial community in topsoil is highly vulnerable to the influence of plant rhizosphere exudates and the ground environment, which drives its diversity and function in maintaining the stability of the community structure [43]. Moreover, root exudates, especially organic acids such as oxalic, malic, and citric acids, improve the solubility of soil nutrients (K, Fe, and P) through acid hydrolysis and double decomposition [40, 44, 45]. This process is especially important in nutrient-poor ecosystems, such as desert soils. Additionally, the number of unique bacterial species in the rhizosphere of *N. atacamensis* was higher than that in the bulk soil. This may be because plant type is the major factor that influences bacterial community distribution [12, 35–37, 44–46], and the plant rhizosphere could recruit specific bacterial populations [14]. However, as this is the first report of *N. atacamensis* rhizosphere characterization, the reason for its higher diversity than that of the bulk soil may only be hypothesized.

### Soil bacterial composition associated with N. atacamensis

The bacterial composition of the bulk soil samples was dominated by Pseudomonadota, Bacillota, and Actinomycetota, especially in samples from the Aguas de Quelana hydrogeological system. However, not all sampling sites had the same phylum distribution. On Puilar, Vega los Pantanos, and Vegas de Cas, Bacillota showed a lower relative abundance whereas Planctomycetota and Chloroflexota became relevant. The presence of Chloroflexota is consistent with previous findings focused on bacterial community variations at the surface and subsurface of harsh environments [47, 48]. This phylum and Actinomycetota dominate soil microbial communities in the hyper-arid margin of the Atacama Desert, with extremely low abundance of Acidobacteria and Alpha- and Betaproteobacteria [49]. Additionally, other soils of the Atacama Desert are mostly dominated by Actinomycetota, Chloroflexota, Pseudomonadota, and Gemmatimonadetes [12, 50, 51]. Although Planctomycetota is detected in extreme ecosystems, it is commonly associated both with plants [12, 52] and bulk soil [53]. All bulk soil samples collected from the Aguas de Quelana hydrogeological system were closely related in terms of the composition and abundance of microorganisms, even when the physicochemical composition did not group them together. This implies that the chemical composition is less relevant for microbial diversity, and the mechanism associated with the structure and behavior of the Aguas de Quelana system is more relevant for the distribution of microorganisms at these sampling points. Since Aguas de Quelana and Soncor systems have different behavior in terms of vegetation distribution and hydrological interconnection, it is possible to hypothesize which of these characteristics has a greater impact on how microbial populations are generated and shared. The Aguas de Quelana system has a continuum of vegetation between all sampling points, which suggests that the homogenization of microorganisms occurs due to the interaction with wild animals or domestic livestock [54]. In contrast, the Soncor system has a greater hydrological connection (and much less vegetation). Therefore, the homogenization of microorganisms could only occur during flooding events, which is unusual and only occurs in years of high rainfall [55]. The rhizosphere samples were predominately composed of almost the same phyla as in the bulk soil; however, certain samples displayed specific trends. In Vegas Carvajal samples, the predominant taxon was Planctomycetota, followed by Pseudomonadota. Actinomycetota was the predominant phylum in Aguas de Quelana 2 and Vegas de Cas, whereas Bacillota represented more than half the total relative abundance in Aguas de Quelana 1 samples. This indicates that the distribution of the most predominant phyla and their relative abundances showed differences between samples, even at the phylum level. This is consistent with the clustering of rhizosphere samples, which did not allow the grouping of the samples in the same distribution as those of the bulk soil samples, implying different underlying bacterial selection and proliferation mechanisms. The distribution was heterogeneous at the genus level, although several taxa were common among all samples. The *Bacillus* genus was the most prevalent in all samples, which is consistent with its role in the rhizosphere [56]. The *Bacillus* genus is a major type of rhizobacteria that forms spores and survives in the soil for long periods under adverse environmental conditions [56]. However, *Bacillus* was not the predominant genus in all samples. For example, the Pir4 lineage was the most prevalent genus in both Vegas Carvajal and Aguas de Quelana 3 samples from the Aguas de Quelana System, and in the samples from Puilar, which belongs to the Soncor System. This result is notable because, among the 20 most abundant genera, the Pir4 lineage is the only one that belongs to the phylum Plancotmycetota and is present in many different habitats, such as oceans, marine sediments, freshwater lakes, sewage, and terrestrial soils. Anaerobic autotrophs of this phylum oxidize ammonia to N_2_ without oxygen; thus, they may play a key role in the global nitrogen cycle [57]. Planctomycete diversity is higher in anoxic soils than in oxic rhizospheres, suggesting that changes in soil oxygen distribution affect the Planctomycetes community [58]. This could be related to the water dynamics of the salt flats, where the water levels change according to factors such as precipitation and evaporation [3]. Additionally, the Pir4 lineage has been described as part of the core microbiome of semi-arid plants [59]. Furthermore, the genus *Candidatus* Tremblaya, which has been described exclusively as a symbiont of mealybugs, was detected only in the Vegas Tarar samples. Given its obligatory intracellular niche and extensive reliance on the host insect for critical metabolic function, this genus cannot survive independently in soil or plant-associated environments [60]. Thus, its presence in rhizosphere samples suggests the presence of mealybugs or related insects that harbor this endosymbiont. Consequently, detection of *Candidatus* Tremblaya could reflect insect infestation in the plant’s immediate environment, rather than an independent soil or plant bacterium. *Pseudococcus* is the most common mealybug genus in Chile. However, its detection is yet to be reported on Salar de Atacama, even with several species detected across the country (including the Atacama region) [61].

The analysis of differential abundance showed differences between the rhizosphere and bulk soil samples at the phylum and genus levels. Bacteroidetes colonize all plant compartments, including the phyllosphere (aerial parts of the plant), rhizosphere (soil-root interface), and root endosphere (inner root tissue) [62]. Bacteroidetes grow in the presence of high carbon concentrations; furthermore, they typically serve as symbionts for plant species [62]. Several genera that are commonly associated with the rhizosphere were overrepresented in the rhizosphere samples. These include *Bacillus*, Pir4 lineage, *Nesterenkonia* [63], *Sinomicrobium* [64], *Salegentibacter* [65], *Pelagibacterium* [38], *Glycomyces* [66], *Woeseia* [67], *Luteolibacter* [68], *Azoarcus* [69], *Jiangella* [70], *Isoptericola* [71], and *Promicromonospora* [72]. Some strains of these genera promote plant growth [12, 36, 37, 43, 65].

### Selective microbial recruitment in the rhizosphere of *N. atacamensis* beyond geographical influence

The structure of the bacterial profile in the rhizosphere samples was not related to the geographical location. Therefore, the plant likely recruits microorganisms that can help it survive the difficult conditions typical of the Salar de Atacama. Although this behavior is widely known [45, 73, 74], geographical and climatic conditions sometimes modulate the effects of soil properties on rhizosphere microbial diversity, thereby affecting the driving effect of microbial diversity on soil multifunctionality [75, 76]. In *N. atacamensis* rhizosphere samples, almost no correlation was observed with geography except in clade 1, which had two samples from the Aguas de Quelana system and clustered with Puilar (Soncor system) and Vega los Pantanos (the northern sampling site). Furthermore, geographical distance negatively affects the similarity between widely separated groups of plants, even in plants of the same species [77]. This may be especially relevant in *N. atacamansis* because the isolation of the populations has led to the hypothesis that they may be genetically distinct [8, 78], which facilitates the diversification of their rhizosphere communities. Our results suggest that the bulk soil, but not the rhizosphere of the Aguas de Quelana System, contains a homogeneous distribution of microorganisms; based on this, we hypothesized that these populations have particular needs that are likely associated with the physicochemical conditions of each soil type. The clustering of the rhizosphere samples (Fig. 6A) showed a different structure from that of the sites grouped according to physicochemical parameters (Fig. 1), suggesting that only some of these parameters are important for the selection of microorganisms. This further suggests that non-considered chemical factors could also have an impact, implying an ecological mechanism.

### Bacterial diversity could be related to microhabitat conditions

The identification of three clades was consistent with the beta diversity analyses, showing similar organization and significant differences between populations, from phylum to genus levels (Fig. 6A). Although these clades contained several shared microorganisms (144 genera in total), they also contained unique genera (196 in clade 1, 80 in clade 2, and 68 in clade 3). The clustering did not correspond to geographical proximity; rather, it corresponded to microhabitat conditions. Although all sites presented saline soil (Fig. 1), different patterns of salt accumulation were observed on each clade. Similar behavior was reported in a previous study examining bulk soil in the Great Salt Plains in Oklahoma [76]. Samples obtained from a visible salt crust, with high salinity and low moisture, were dominated by oxygenic cyanobacteria such as *Cyanothece* and *Euhalothece*, which are adapted to oxidized and evaporative environments. In contrast, soils without surface salt crusts and with higher moisture harbored anaerobic sulfur-cycling bacteria such as *Desulfuromonas*, *Halochromatium*, and *Thiohalorhabdus*. This contrast indicates that surface salinity patterns reflect deeper microhabitat differences that structure microbial communities.

Sampling points grouped in clade 1 (Pular, Aguas de Quelana 3, Vegas Carvajal, and Vega los Pantanos) exhibited visible salt crusts or accumulations on the surface, indicating higher evaporation rates and a seasonally variable water table, which likely results in periods of greater soil moisture near the surface [3, 4]. These sites also shared several bacterial genera, belonging mainly to the phyla Pseudomonadota, Planctomycetota, and Chloroflexota, which have been detected in hypersaline ecosystems [79]. Pseudomonadota, which are renowned for their metabolic versatility and adaptive capacity, typically flourish in environments where moisture is readily available to support their copiotrophic lifestyles [79]. In contrast, the presence of Planctomycetota (Pir4 lineage) and Chloroflexota (particularly those within the class Dehalococcoidia, S085, and SAR202) suggests that they facilitate plant growth and proliferation when water levels reach the rhizosphere. A recent report identified Planctomycetota as a pioneer taxon in root colonization following artificial irrigation, a condition similar to the intermittent presence of water at these sites [80]. Additionally, Chloroflexota is attracted by root exudates of maize and is involved in host-medicated rhizosphere microbiota assembly, consequently promoting the growth and defense capability of the host [81]. Thus, the ecological function on this ecosystem could be similar.

Clade 2 (Vegas de Cas, Aguas de Quelana 2, and Vegas Tarar) included sites with saline soil but without visible surface salt deposits, suggesting deeper water tables and lower surface moisture. These conditions were associated with higher abundances of genera belonging to the phyla Actinomycetota and Bacteroidota. The phylum Actinomycetota is commonly associated with drier and more stable saline soil, even in desert ecosystems [13]. The slower growth rate and higher drought tolerance could favor the survival of this phylum and allow it to colonize plant roots [13, 82]. Additionally, many genera belonging to this phylum can sporulate to survive, which confers another adaptative advantage under adverse conditions [83]. An increase in the abundance of Bacteroidota has been reported in dry environments. In hot deserts, the humidity level affects the diversity of endophytic bacteria, with Actinomycetota and Bacteroidota exhibiting increased abundance in dry periods [82]; this is consistent with our results. The Bacteroidota phylum plays a crucial role in both host-microbe interactions and broader ecosystem functioning. In particular, it is involved in global nutrient cycling, the breakdown of complex organic matter, and promotion of plant growth. Moreover, this phylum is commonly associated with water presence. Thus, the plant could be recruiting these bacteria to improve its fitness [62].

The diversity of specific bacteria in the rhizosphere of different *N. atacamensis* populations reinforces the importance of the species as a habitat, making these microbes relevant to its conservation. This demonstrates the need for continued microbial biodiversity in the desert. Among all the sampling points, only two sites from clade 1 (Vega los Pantanos and Vega Carvajal) and Aguas de Quelana 1 (clade 3) are located within the protected area of the ‘Reserva Nacional Los Flamencos’. Although several of the remaining sites are recognized as biodiversity priority areas in the regional conservation strategy, none of the clade 2 locations benefit from official protection against extraction, disturbance, or land use change. This represents a significant threat to the natural biodiversity associated with *N. atacamensis*, a species already classified as endangered.

### Association between genus presence and physicochemical variables

Soil salinity was high throughout the soil profile, as reflected by the EC (Fig. 1). This seems to shape bacterial communities owing to intense selective pressure because only a few bacteria can develop over large salt concentration gradients [76]. The EC values were exceptionally high (approximately 24.5–151 dS m^–1^) in the surface zone. However, ions, but not EC, had a significant effect on the bacterial community structure and showed correlation with some of the genera detected in the rhizosphere (Fig. 7 and Supplementary Fig. 10). The genera *Salipaludibacillus*, *Bacillus*, and MB-A2-108 showed a positive correlation with chloride, calcium, potassium, and magnesium, indicating the presence of highly tolerant bacteria. Although halotolerant bacteria are commonly found in extreme environments such as deserts [12, 15, 47, 49, 79] or commonly isolated from halophytic plants [84], the correlation between some of the genera and the presence of chloride (which is commonly associated with halophytes and salt tolerance) is expected. This suggests that plants with higher chloride levels tend to recruit specialized halophilic bacteria, most likely to mitigate the osmotic pressure of this ion. Additionally, some of the families present in the soil profile include halophilic taxa or are present in other saline environments, such as Pseudomonadota (*Comamonadaceae*, *Marinobacteraceae*, *Nitrosomonadaceae*, and *Pseudomonadaceae*), Actinomycetota (*Bifidobactericeae* and *Sporichthyaceae*), and Bacillota (*Bacilliaceae* and *Salisediminibacteriaceae*), which have been detected in different saline environments [85]. Members of the families Chitinophagaceae, Ilumatobacteraceae, Rubritaleaceae, Pedosphaeraceae, and Pirellulaceae have been detected in various soils and sediments such as desert surfaces (China), arid biological crusts (United States), and hypersaline lagoons (Chile) [15, 86–90]. *Salipaludibacillus* is related positively to chlorides but negatively with pH, which is contrary to the alkalophilic nature of this genus and its moderately halophilic strains. Both *Bacillus* and *Salipaludibacillus* are members of Bacillota phylum, which does not present differences across the rhizosphere clades. This implies that these genera have an important role in plant development. Their role may be conferred through the solubilization of minerals — such as calcium, potassium, and magnesium, with which they are correlated — thereby increasing the availability of nutrients to crops [91].

Nitrate-solubilizing bacteria can convert nitrate (NO_3_^−^) into other nitrogenous compounds, such as nitrite (NO_2_^−^), nitric oxide (NO), nitrous oxide (N_2_O), or even nitrogen gas (N_2_), through a process called denitrification. Our results suggest a strong correlation between nitrate presence and *Sinomicrobium*, *Pelagibius*, and Acidobacteria Subgroup 2. *Sinomicrobium* strains with PGP traits (such as indole-3-acetic acid production) [92] and nitrate conversion capacity [64] have been detected. Although the role of Acidobacteria in nitrogen cycling has been reported, its specific ecological role remains unclear [93, 94]. *Nesterenkonia* and *Aliifodinibius* have been detected under alkalophilic conditions. Thus, our findings are consistent with previous reports [95, 96].

A large group of genera is not directly related to the evaluated chemical variables. Even when there is a negative correlation with potassium, chloride, calcium, and magnesium, none of the variables have a positive relation to their relative abundance. All genera share the same quadrant of clade 1 samples, which present salt-crust on the surface. This leads to higher soil moisture and lower oxygen levels. This is consistent with reports of the anaerobic or facultative anaerobic genus *Woeseia* [97], a Pir4 lineage detected in anoxic or micro-oxic habitats [98]. The uncultured *Rhizobiales* Amb-16 S-1323 has been detected in hypoxic and anaerobic environments [99], and the SAR202 clade has been proposed as a facultative anaerobic [100]. Therefore, the evaluation of oxygen levels in the vicinity of the roots may be the key to determining the mechanisms underlying bacterial recruitment by *N. atacamensis*.

## Conclusion

This study provides the first comprehensive characterization of the rhizosphere and bulk soil bacterial communities associated with the endangered halophyte *N. atacamensis* in the Salar de Atacama. In this study, we identified distinct microbial communities that were more diverse in rhizosphere samples than in bulk soil. Rhizosphere samples clustered into three clades defined by differentially abundant genera, independent of geographic location. Our findings suggest selective microbial recruitment by *N. atacamensis* in response to specific environmental stresses and microhabitat conditions associated with the presence/absence of salt-crusts on the sampling sites. These findings confirm that this species can alter the bacterial biodiversity of soils. This demonstrates the need for further studies of endemic species in all ecosystems wherein the plant develops. Furthermore, these findings highlight the importance of protecting ecological niches where endemic and endangered plants grow and develop.

Bulk soil samples were more homogeneous in the Aguas de Quelana hydrogeological system, which was likely due to vegetation continuity and animal-mediated dispersal, whereas the Soncor system showed greater microbial heterogeneity. These results provide a theoretical basis for elucidation of the regulatory mechanisms of microbial communities in extreme ecosystems. Moreover, the presence of specialized bacterial taxa adapted to salinity and aridity suggests potential for use in sustainable agriculture under stress conditions.

Our results further highlight the need for conservation strategies to preserve *N. atacamensis* and its unique microbiota in the face of increasing anthropogenic and climatic pressures. However, more complex metagenomic studies are required to elucidate the mechanisms through which this plant interacts with its microbiota and survives extreme conditions. The isolation of microorganisms from the *N. atacamensis* rhizosphere and endosphere could help elucidate these mechanisms. Furthermore, the isolated microbes could be subsequently used as a biotechnological input for the growth of other plants that are more susceptible to salt and water stress.

## Supplementary Information

Below is the link to the electronic supplementary material.

**Supplementary table 1 (Part 1)**: Physicochemical characterization of rhizosphere and bulk soils from the sampling sites. The samples are ordered from north to south according to their geographical location. **Supplementary table 1 (Part 2)**: Physicochemical characterization of rhizosphere and bulk soils from the sampling sites. The samples are ordered from north to south according to their geographical location. **Supplementary table 1 (Part 3)**: Physicochemical characterization of rhizosphere and bulk soils from the sampling points. The samples are ordered from north to south according to their geographical location. **Supplementary Figure 1**: Hierarchical clustering dendrogram. Clustering of the sampling sites based on the physicochemical characteristics of the bulk soil. **Supplementary Figure  2**: Alpha diversity for *Nitrophila atacamensis* rhizosphere (sample) and bulk soil (control) based on four indices (Chao1, A; Observed, B; Shannon, C; and Simpson, D). Wilcoxon test, * <0.05, ** <0.01. **Supplementary Figure  3**: Alpha diversity for all and *N. atacamensis* rhizosphere samples based on four indices (Chao1, A; Observed, B; Shannon, C; and Simpson, D). Kruskal–Wallis, *P* > 0.05. **Supplementary Figure  4**: Alpha diversity for all and bulk soil samples based on four indices (Chao1, A; Observed, B; Shannon, C; and Simpson, D). Kruskal–Wallis, *P* > 0.05. **Supplementary Figure  5**: Beta diversity for *Nitrophila atacamensis* rhizosphere (sample) and bulk soil (control) based on the following indices: A) Bray–Curtis (PERMANOVA, *P* = 0.00466), A; Canberra, B (PERMANOVA, *P* = 0.0041); Jaccard, C (PERMANOVA, *P* = 0.00495); JSD, D (ns); Unifrac, E (PERMANOVA, *P* = 0.00579); and Weighted Unifrac, F (ns). **Supplementary Figure  6**: Clustering of bulk soil of sampling sites based on the relative abundance of the phylum. Sites of Soncor and Aguas de Quelana systems are shown as red and blue squares, respectively. **Supplementary Figure  7**: Clustering of the rhizosphere of *N. atacamensis* of sampling sites based on the relative abundance of phyla. Sites of Soncor and Aguas de Quelana systems are shown as red and blue squares, respectively. **Supplementary Figure  8**: Beta diversity for rhizosphere samples. A) Bray–Curtis (PERMANOVA, *P* = 9.99991e-06), A; Canberra, B (PERMANOVA, *P* = 9.99991e-06); Jaccard, C (PERMANOVA, *P* = 9.99991e-06); JSD, D (PERMANOVA, *P* = 9.99991e-06); Unifrac, E (PERMANOVA, *P* = 9.99991e-06); and Weighted Unifrac, F (9.99991e-06). **Supplementary Figure  9**: Differential abundance of exclusively clade 3 genera. Differential abundance of the 11 genera with differences between rhizosphere clades (LEfSe, α = 0.05; Kruskal–Wallis, *** = *p* < 0.001). **Supplementary Figure  10**: Correlations between genus and physicochemical variables. Spearman correlation matrix between all genera with relative abundance > 0.1%, and physicochemical parameters from the rhizosphere samples. Each box shows the Spearman correlation for each taxon (rows) and physicochemical variable (columns)

## Data Availability

All raw sequences have been deposited in the National Center for Biotechnology Information (NCBI) database (Bethesda, MD, USA) under BioProject accession code PRJNA1216253.

## Abbreviations

OM: Organic matter
PCR: Polymerase chain reaction
OTUs: Operational taxonomic units
ECs: Electrical conductivities
RDA: Redundancy analysis
LEfSe: Linear discriminant analysis Effect Size
ANCOM-BC2: Analysis of Compositions of Microbiomes with Bias Correction 2
