# Supplementary material for: Characterization of bacterial diversity associated with a Salar de Atacama native plant Nitrophila atacamensis
Source: Environ Microbiome. 2025 Aug 6;20:100. doi: 10.1186/s40793-025-00766-7 (PMC12329874; doi:10.1186/s40793-025-00766-7)
Supplement: Supplementary file 1 — Supplementary table 1 (Part 1): Physicochemical characterization of rhizosphere and bulk soils from the sampling sites. The samples are ordered from north to south according to their geographical location. Supplementary table 1 (Part 2): Physicochemical characterization of rhizosphere and bulk soils from the sampling sites. The samples are ordered from north to south according to their geographical location. Supplementary table 1 (Part 3): Physicochemical characterization of rhizosphere and bulk soils from the sampling points. The samples are ordered from north to south according to their geographical location. Supplementary Figure 1: Hierarchical clustering dendrogram. Clustering of the sampling sites based on the physicochemical characteristics of the bulk soil. Supplementary Figure 2: Alpha diversity for Nitrophila atacamensis rhizosphere (sample) and bulk soil (control) based on four indices (Chao1, A; Observed, B; Shannon, C; and Simpson, D). Wilcoxon test, * <0.05, ** <0.01. Supplementary Figure 3: Alpha diversity for all and N. atacamensis rhizosphere samples based on four indices (Chao1, A; Observed, B; Shannon, C; and Simpson, D). Kruskal–Wallis, P > 0.05. Supplementary Figure 4: Alpha diversity for all and bulk soil samples based on four indices (Chao1, A; Observed, B; Shannon, C; and Simpson, D). Kruskal–Wallis, P > 0.05. Supplementary Figure 5: Beta diversity for Nitrophila atacamensis rhizosphere (sample) and bulk soil (control) based on the following indices: A) Bray–Curtis (PERMANOVA, P = 0.00466), A; Canberra, B (PERMANOVA, P = 0.0041); Jaccard, C (PERMANOVA, P = 0.00495); JSD, D (ns); Unifrac, E (PERMANOVA, P = 0.00579); and Weighted Unifrac, F (ns). Supplementary Figure 6: Clustering of bulk soil of sampling sites based on the relative abundance of the phylum. Sites of Soncor and Aguas de Quelana systems are shown as red and blue squares, respectively. Supplementary Figure 7: Clustering of the rhizosphere of N. atacamensis of sampling sites based o [file 40793_2025_766_MOESM1_ESM.docx]

**Title**

**Characterization of bacterial diversity associated with a Salar de Atacama native plant *Nitrophila atacamensis***

**Supplementary Tables**

**Supplementary Table 1 (Part 1).** Physicochemical characterization of rhizosphere and bulk soils from the sampling sites. The samples are ordered from north to south according to their geographical location.

|  |  |  | Coordinates (UTM 19S) | | | Physicochemical parameters | | |
| --- | --- | --- | --- | --- | --- | --- | --- | --- |
| Site | Code | Sample type | South (m) | Estern (m) | Altitude (m) | EC (dS/m) | pH | Phosphates (mg/L) |
| Los Pantanos Poniente | VPN | Rhizosphere | 7,446,800 | 592,251 | 2.355 | 62.50 | 7.70 | 2.92 |
|  | VPT | Bulk soil |  |  |  | 88.50 | 8.02 | 2.92 |
| Vegas de Tarar | VTN | Rhizosphere | 7,430,898 | 591,163 | 2.334 | 90.00 | 8.44 | 2.92 |
|  | VTT | Bulk soil |  |  |  | 52.60 | 6.89 | 2.92 |
| Puilar | PUN | Rhizosphere | 7,424,521 | 588,943 | 2.317 | 138.00 | 8.76 | 2.92 |
|  | PUT | Bulk soil |  |  |  | 88.30 | 9.12 | 9.82 |
| Vegas Carvajal | VCN | Rhizosphere | 7,411,745 | 593,079 | 2.311 | 40.10 | 7.78 | 2.92 |
|  | VCT | Bulk soil |  |  |  | 121.00 | 8.53 | 4.66 |
| Aguas de Quelana 1 | AQN1 | Rhizosphere | 7,409,557 | 592,658 | 2.303 | 151.00 | 8.22 | 2.92 |
|  | AQT1 | Bulk soil |  |  |  | 165.00 | 8.54 | 2.92 |
| Aguas de Quelana 2 | AQN2 | Rhizosphere | 7,405,844 | 594,714 | 2.273 | 60.60 | 8.47 | 2.92 |
|  | AQT2 | Bulk soil |  |  |  | 255.00 | 8.61 | 2.92 |
| Aguas de Quelana 3 | AQN3 | Rhizosphere | 7,403,520 | 594,461 | 2.309 | 24.50 | 8.36 | 2.92 |
|  | AQT3 | Bulk soil |  |  |  | 46.70 | 7.61 | 377 |
| Vegas de Cas | VKN | Rhizosphere | 7,401,234 | 603,402 | 2.599 | 128.00 | 8.71 | 3.40 |
|  | VKT | Bulk soil |  |  |  | 297.00 | 8.71 | 4.60 |

**Supplementary Table 1 (Part 2).** Physicochemical characterization of rhizosphere and bulk soils from the sampling sites. The samples are ordered from north to south according to their geographical location.

|  | Physicochemical parameters | | | | | | |
| --- | --- | --- | --- | --- | --- | --- | --- |
| Site | Sulfates (mg/L) | Nitrates (mg/L) | Chlorides (mg/L) | Ammonium (mg/L) | Calcium (mg/L) | Magnesium (mg/L) | Potassium (mg/L) |
| Los Pantanos poniente | 191.00 | 0.16 | 389.00 | 0.28 | 31.80 | 14.40 | 21.60 |
|  | 277.00 | 0.44 | 533.00 | 0.28 | 29.30 | 7.95 | 53.20 |
| Vegas de Tarar | 294.00 | 0.47 | 530.00 | 0.28 | 20.20 | 1.36 | 67.90 |
|  | 113.00 | 1.15 | 381.00 | 0.28 | 32.70 | 6.21 | 38.20 |
| Puilar | 431.00 | 0.22 | 920.00 | 0.28 | 5.62 | 11.20 | 136.00 |
|  | 330.00 | 0.16 | 525.00 | 0.28 | 0.45 | 9.58 | 73.80 |
| Vegas Carvajal | 332.00 | 0.68 | 48.00 | 0.28 | 24.50 | 189.00 | 7.72 |
|  | 1,024.00 | 0.16 | 233.00 | 0.30 | 33.20 | 794.00 | 43.20 |
| Aguas de Quelana 1 | 363.00 | 0.16 | 1,009.00 | 0.28 | 54.80 | 254.00 | 95.70 |
|  | 314.00 | 0.50 | 1,271.00 | 0.28 | 74.70 | 354.00 | 102.00 |
| Aguas de Quelana 2 | 202.00 | 0.17 | 373.00 | 0.28 | 46.90 | 94.90 | 27.20 |
|  | 893.00 | 0.34 | 1,753.00 | 0.28 | 40.40 | 335.00 | 123.00 |
| Aguas de Quelana 3 | 45.10 | 0.55 | 220.00 | 0.28 | 5.41 | 9.00 | 23.80 |
|  | 124.00 | 1.57 | 323.00 | 0.28 | 68.30 | 41.60 | 35.90 |
| Vegas de Cas | 238.00 | 0.88 | 321.00 | 0.28 | 53.90 | 8.67 | 23.80 |
|  | 1,572.00 | 9.68 | 1,467.00 | 0.28 | 34.10 | 1.15 | 76.60 |

**Supplementary Table 1 (Part 3).** Physicochemical characterization of rhizosphere and bulk soils from the sampling points. The samples are ordered from north to south according to their geographical location.

|  | Physicochemical parameters | | | |
| --- | --- | --- | --- | --- |
| Site | Sodium (mg/L) | Boron (mg/L) | Bicarbonate (mg/L) | Organic matter (%) |
| Los Pantanos poniente | 575.00 | 73.00 | 0.18 | 0.75 |
|  | 871.00 | 83.90 | 2.26 | 0.58 |
| Vegas de Tarar | 886.00 | 102.00 | 6.64 | 0.83 |
|  | 443.00 | 2.20 | 0.16 | 0.65 |
| Puilar | 1,380.00 | 1,213.00 | 0.34 | 1.84 |
|  | 883.00 | 3,150.00 | 14.60 | 2.97 |
| Vegas Carvajal | 184.00 | 67.50 | 1.43 | 0.25 |
|  | 480.00 | 252.00 | 0.29 | 0.32 |
| Aguas de Quelana 1 | 1,303.00 | 173.00 | 0.29 | 0.94 |
|  | 1,217.00 | 441.00 | 0.94 | 0.54 |
| Aguas de Quelana 2 | 464.00 | 321.00 | 0.16 | 1.46 |
|  | 2,395.00 | 604.00 | 0.16 | 0.7 |
| Aguas de Quelana 3 | 261.00 | 113.00 | 0.16 | 3.99 |
|  | 353.00 | 335.00 | 2.53 | 5.82 |
| Vegas de Cas | 531.00 | 181.00 | 0.65 | 1.77 |
|  | 3184.00 | 369.00 | 1.17 | 0.28 |

**Supplementary Figures**

**
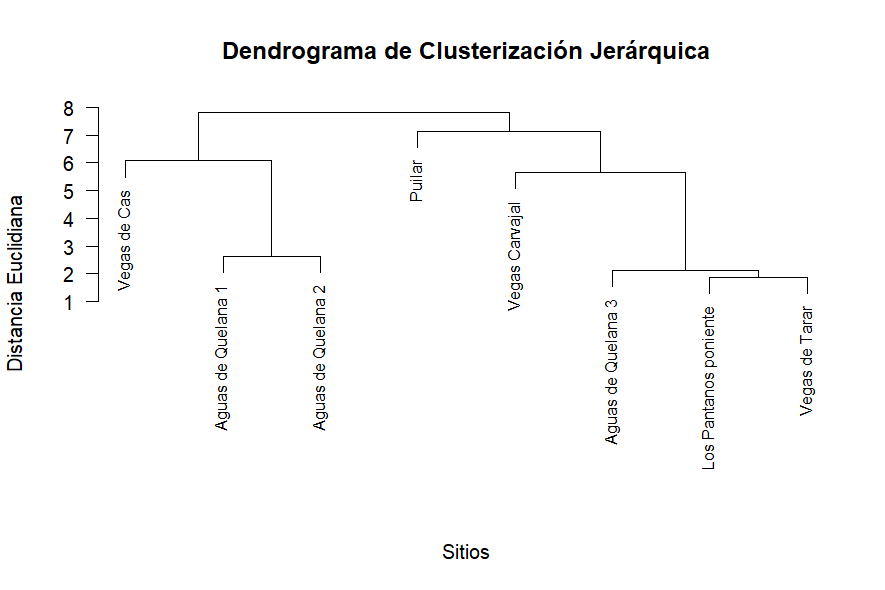
**

**Supplementary Figure 1. Hierarchical clustering dendrogram.** Clustering of the sampling sites based on the physicochemical characteristics of the bulk soil.

**
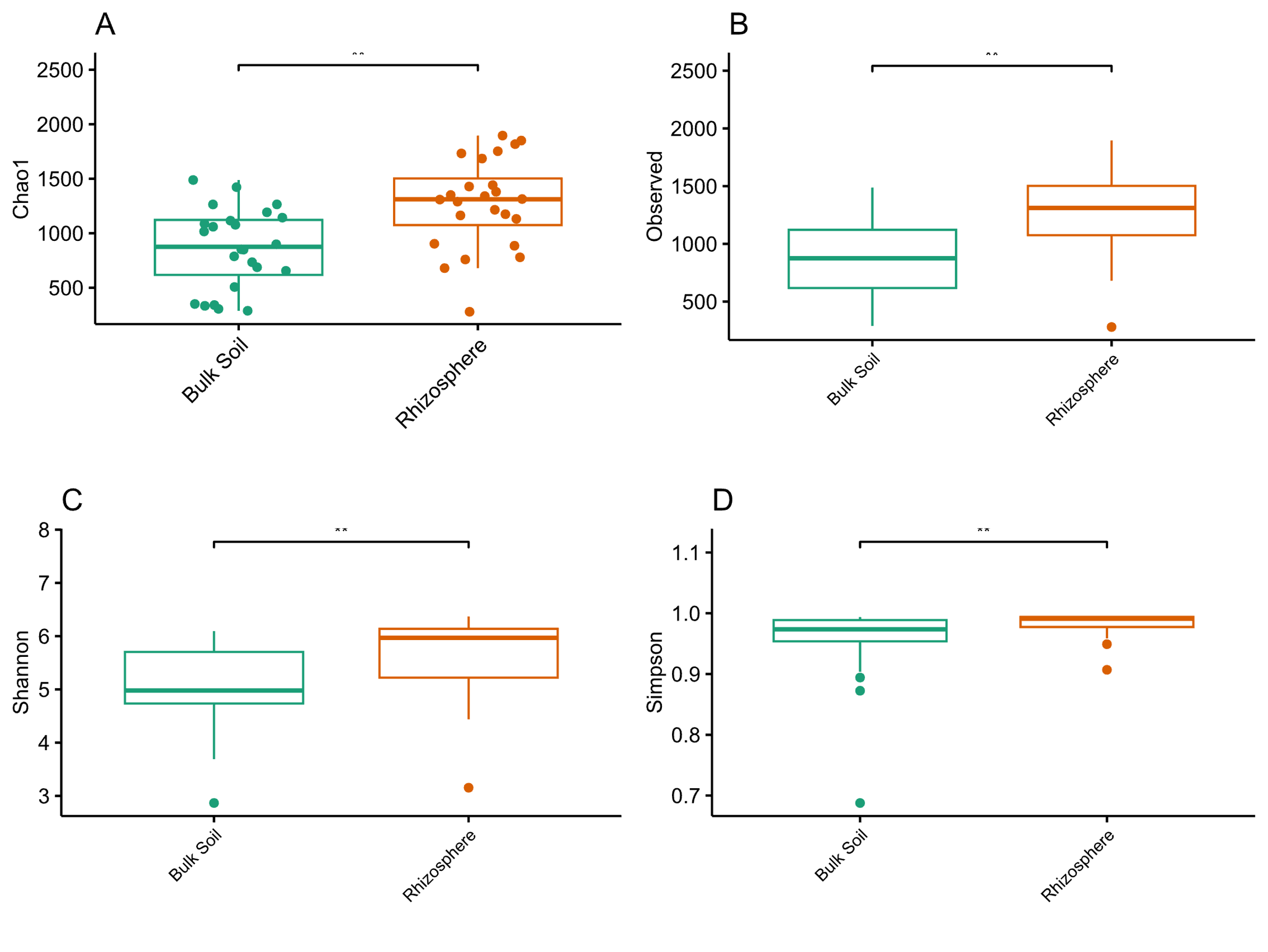
**

**Supplementary Figure 2.** Alpha diversity for *Nitrophila atacamensis* rhizosphere (sample) and bulk soil (control) based on four indices (Chao1, A; Observed, B; Shannon, C; and Simpson, D). Wilcoxon test, *** <0.05, ** <0.01.


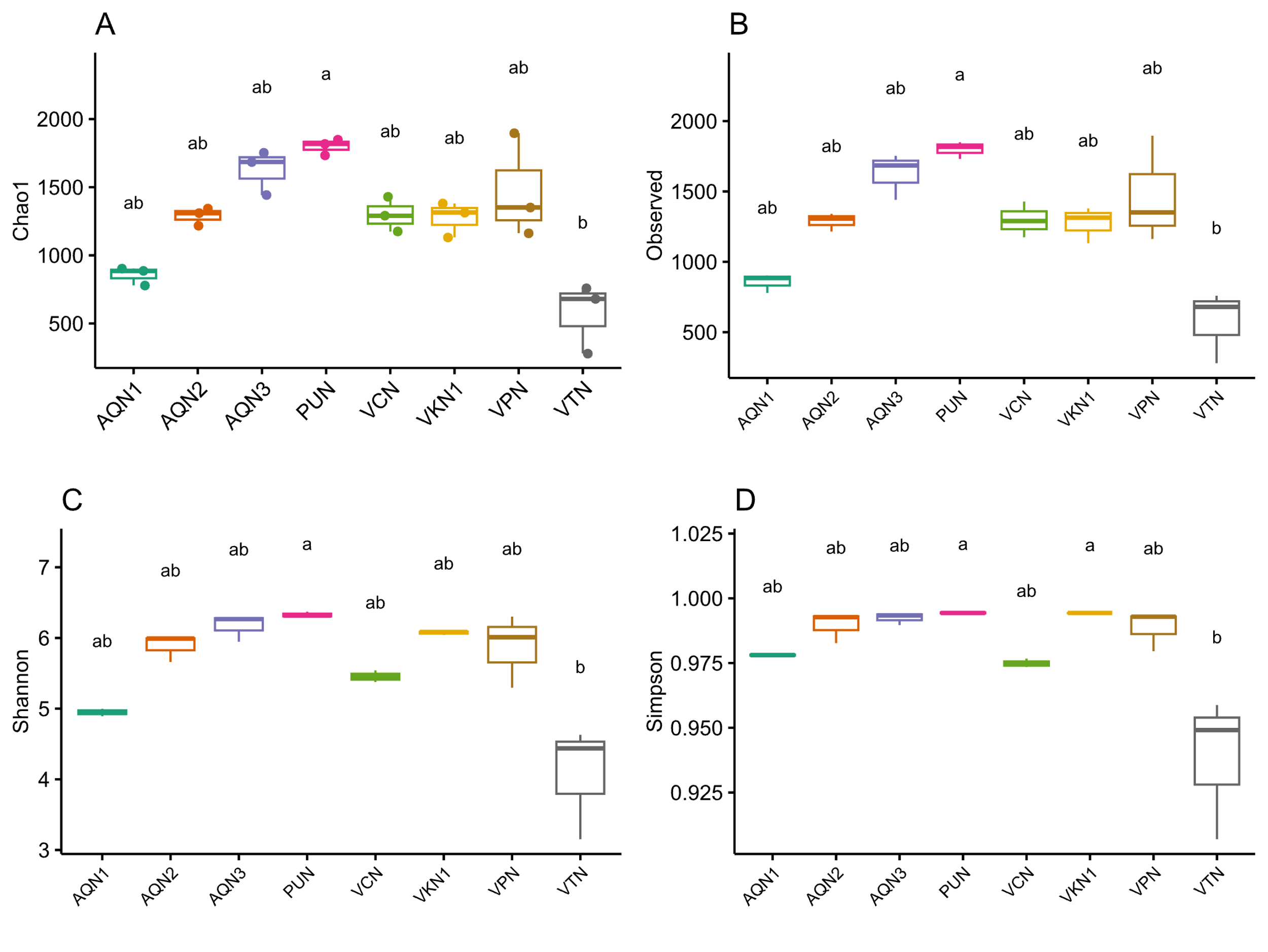


**Supplementary Figure 3.** Alpha diversity for all and *N. atacamensis* rhizosphere samples based on four indices (Chao1, A; Observed, B; Shannon, C; and Simpson, D). Kruskal–Wallis, *P* > 0.05.


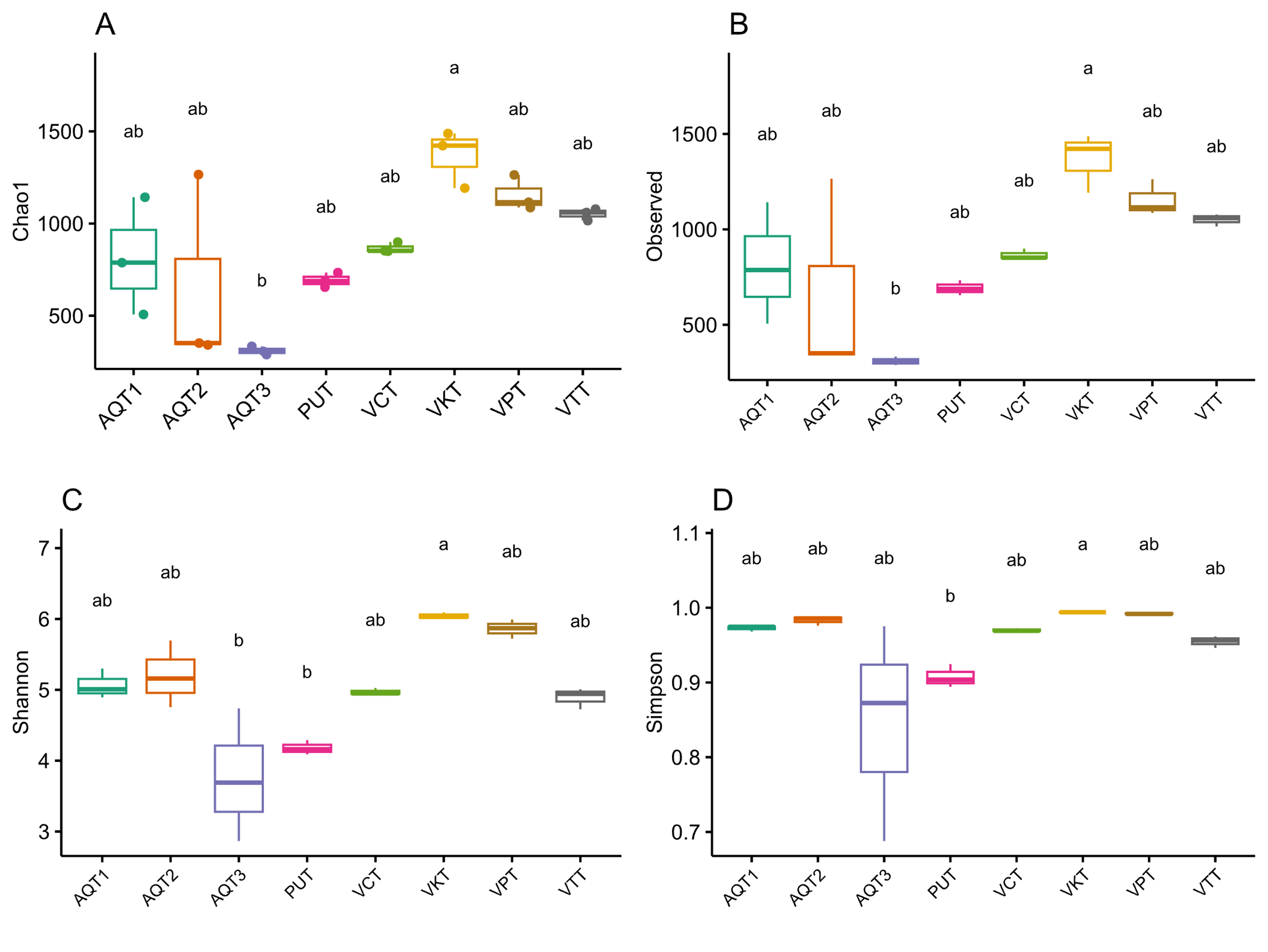


**Supplementary Figure 4.** Alpha diversity for all and bulk soil samples based on four indices (Chao1, A; Observed, B; Shannon, C; and Simpson, D). Kruskal–Wallis, *P* > 0.05.


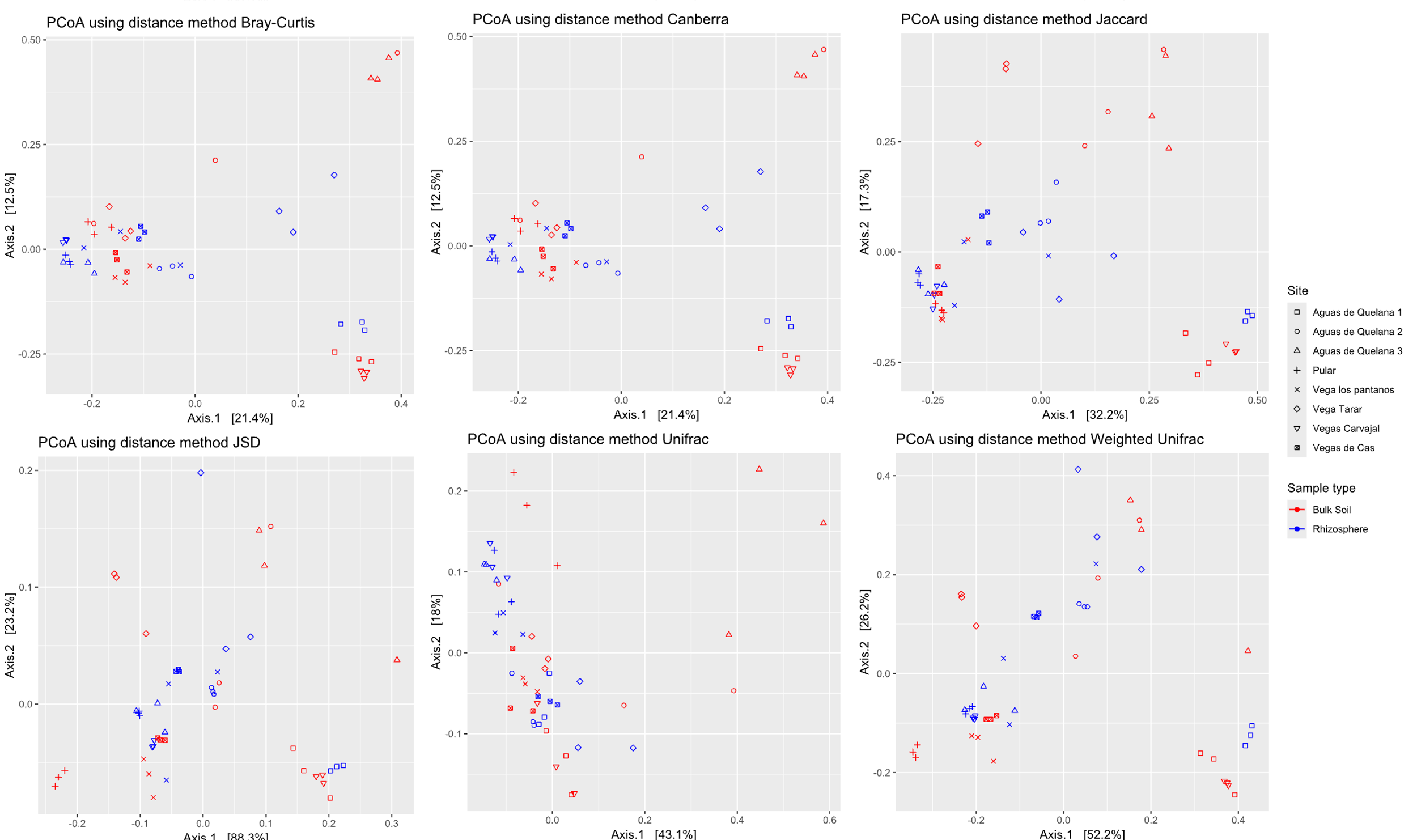


**Supplementary Figure 5.** Beta diversity for *Nitrophila atacamensis* rhizosphere (sample) and bulk soil (control) based on the following indices: A) Bray–Curtis (PERMANOVA, P = 0.00466), A; Canberra, B (PERMANOVA, P = 0.0041); Jaccard, C (PERMANOVA, P = 0.00495); JSD, D (ns); Unifrac, E (PERMANOVA, P = 0.00579); and Weighted Unifrac, F (ns).


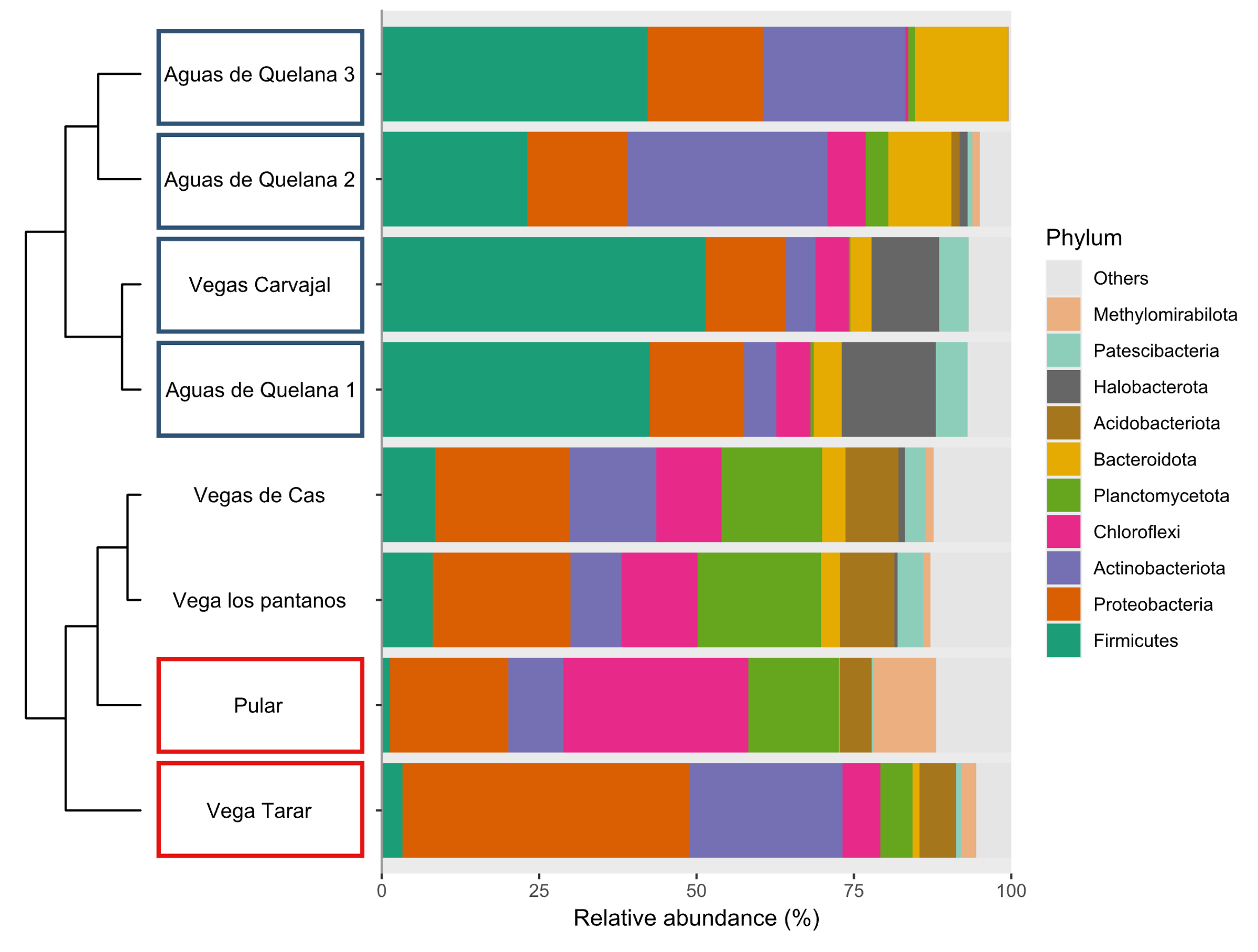


**Supplementary Figure 6**. Clustering of bulk soil of sampling sites based on the relative abundance of the phylum. Sites of the Soncor and Aguas de Quelana systems are shown as red and blue squares, respectively.


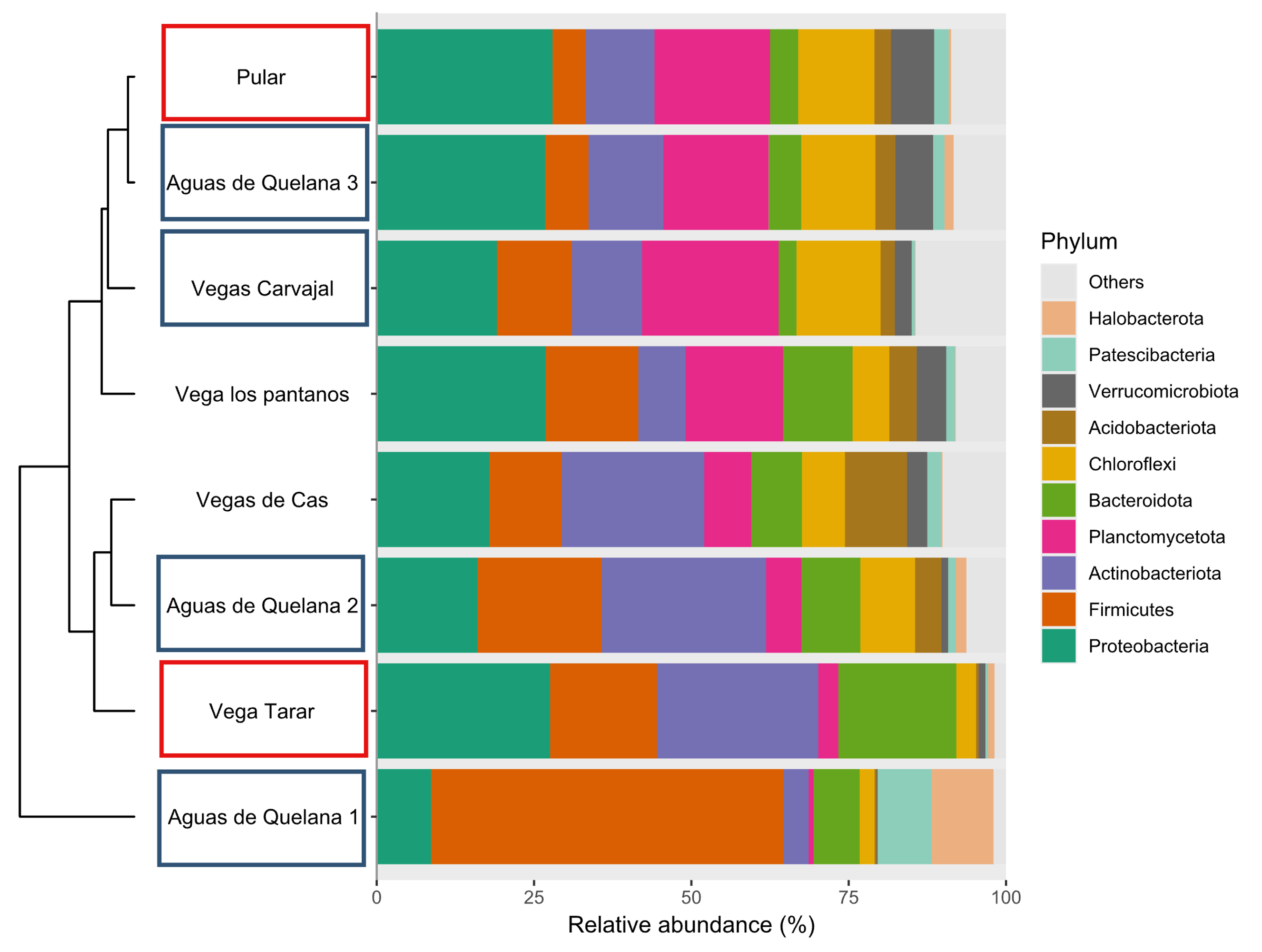


**Supplementary Figure 7**. Clustering of rhizosphere of *N. atacamensis* of sampling sites based on the relative abundance of phyla. Sites of the Soncor and Aguas de Quelana systems are shown as red and blue squares, respectively.


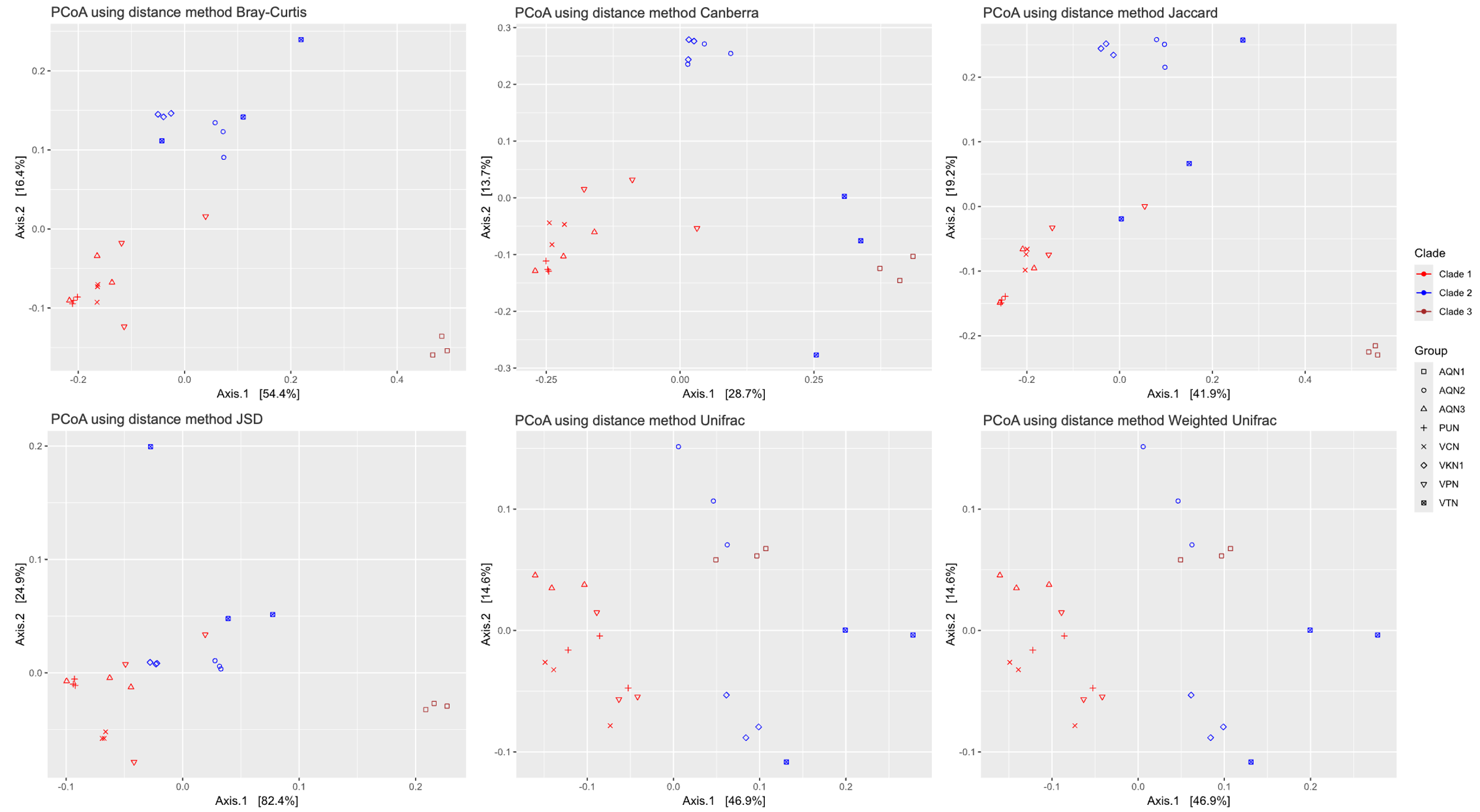


**Supplementary Figure 8**. Beta diversity for rhizosphere samples. A) Bray–Curtis (PERMANOVA, P = 9.99991e-06), A; Canberra, B (PERMANOVA, P = 9.99991e-06); Jaccard, C (PERMANOVA, P = 9.99991e-06); JSD, D (PERMANOVA, P = 9.99991e-06); Unifrac, E (PERMANOVA, P = 9.99991e-06); and Weighted Unifrac, F (9.99991e-06).


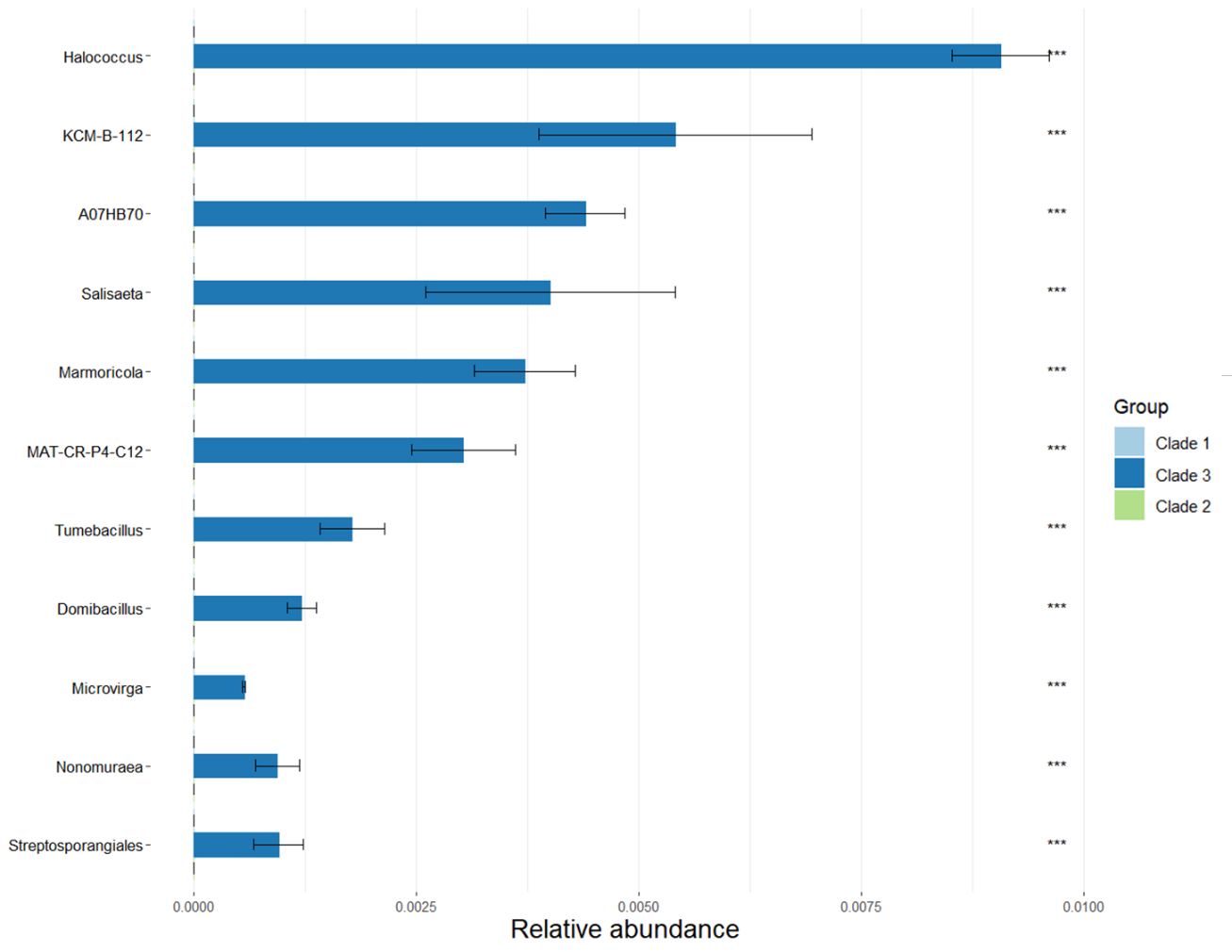


**Supplementary Figure 9. Differential abundance of exclusively clade 3 genera.** Differential abundance of the 11 genera with differences between rhizosphere clades (LEfSe, α = 0.05; Kruskal–Wallis, *** = p < 0.001).


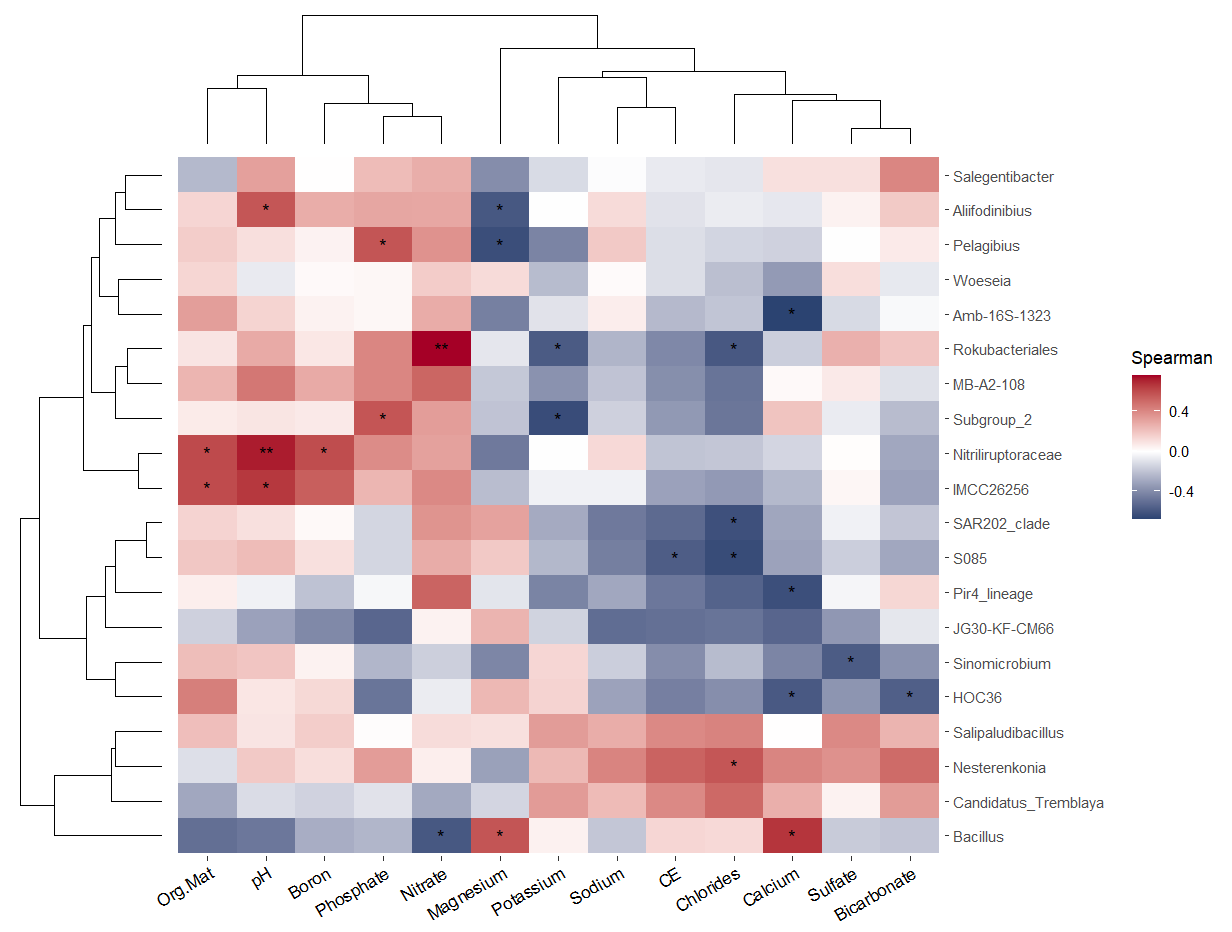


**Supplementary Figure 10**. **Correlations between genus and physicochemical variables.** Spearman correlation matrix between all genera with relative abundance >0.1%, and physicochemical parameters of the rhizosphere samples. Each box shows the Spearman correlation for each taxon (rows) and physicochemical variable (columns).
